# Functional and molecular characterization of the Atlantic salmon gill epithelium cell line ASG-10; a tool for *in vitro* gill research

**DOI:** 10.3389/fmolb.2023.1242879

**Published:** 2023-10-17

**Authors:** Orla Slattery, Maria K. Dahle, Arvind Y. M. Sundaram, Barbara F. Nowak, Mona C. Gjessing, Anita Solhaug

**Affiliations:** ^1^ Marine and Freshwater Research Centre, Atlantic Technological University, Galway, Ireland; ^2^ Norwegian Veterinary Institute, Oslo, Norway; ^3^ Department of Medical Genetics, Oslo University Hospital, Oslo, Norway; ^4^ Institute of Marine and Antarctic Studies, University of Tasmania, Hobart, TAS, Australia

**Keywords:** gill epithelium, ASG-10, Atlantic salmon, cytochromeP450, tight junctions, ABC transporters, transcriptome, proteome

## Abstract

Fish gills are not only the respiratory organ, but also essential for ion-regulation, acid-base control, detoxification, waste excretion and host defense. Multifactorial gill diseases are common in farmed Atlantic salmon, and still poorly understood. Understanding gill pathophysiology is of paramount importance, but the sacrifice of large numbers of experimental animals for this purpose should be avoided. Therefore, *in vitro* models, such as cell lines, are urgently required to replace fish trials. An Atlantic salmon gill epithelial cell line, ASG-10, was established at the Norwegian Veterinary institute in 2018. This cell line forms a monolayer expressing cytokeratin, e-cadherin and desmosomes, hallmarks of a functional epithelial barrier. To determine the value of ASG-10 for comparative studies of gill functions, the characterization of ASG-10 was taken one step further by performing functional assays and comparing the cell proteome and transcriptome with those of gills from juvenile freshwater Atlantic salmon. The ASG-10 cell line appear to be a homogenous cell line consisting of epithelial cells, which express tight junction proteins. We demonstrated that ASG-10 forms a barrier, both alone and in co-culture with the Atlantic salmon gill fibroblast cell line ASG-13. ASG-10 cells can phagocytose and express several ATP-binding cassette transport proteins. Additionally, ASG-10 expresses genes involved in biotransformation of xenobiotics and immune responses. Taken together, this study provides an overview of functions that can be studied using ASG-10, which will be an important contribution to *in vitro* gill epithelial research of Atlantic salmon.

## 1 Introduction

As the demand for food increases, aquaculture is now providing a greater proportion of fish for human consumption than wild fisheries, with Atlantic salmon (*Salmo salar L*) being one of the main farmed fish species (Seafish, 2020). Due to this dependence on fish farming, it is important to ensure fish health and animal welfare in aquaculture. However, despite a lot of progress, some fish losses still occur. For example, according to the Norwegian fish health report of 2022, 56.7 million (16.1%) of the farmed Atlantic salmon died after sea transfer in 2022 (Sommerset et al., 2023). The causes of these deaths were many, with gill diseases being part of the explanation. We cannot address issues with gill diseases without good gill models, which are needed to increase the understanding of Atlantic salmon biology.

Gills are a multifunctional organ responsible for gas-exchange and crucial in ion regulation, detoxification of xenobiotics, excretion of waste products and immunity. The gill surface is in direct contact with water and is thus prone to many water-borne agents. Non-infectious agents, such as pharmaceuticals (Chang et al., 2019), environmental toxins (Evans, 1987), toxic microalgae (John et al., 2022) or pollutants and other water quality issues (Sommerset et al., 2023) may compromise the gills and pave the way for infections. Causes of gill disease in Atlantic salmon are diverse with both non-infectious and infectious etiology (Gjessing et al., 2015; Weli et al., 2017; Herrero et al., 2018; Marcos-Lopez Rodger, 2020; Gjessing et al., 2021). The term “complex gill disease” is used to describe gill disease manifestation with a complex histopathological pattern and is often suspected to have multifactorial etiology.

The functional units of the gill are filaments extending in two rows from the gill arch. The filaments have a cartilage core and support rows of thin closely stacked respiratory units - plate-like lamellae, on the ventral and dorsal side of the filament. The lamellae consist of a network of vascular spaces delineated by modified endothelial cells (pillar cells) and covered by sheets of epithelial cells (pavement cells) (Evans et al., 2005). The thin epithelial layer covering the lamella and the counter current flow of blood and water allows efficient removal of carbon dioxide and uptake of oxygen. Further, a number of mitochondria-rich specialized epithelial cells (chloride cells) regulating the chloride and sodium levels, goblet (mucous) cells and neuroepithelial cells are present in the filament epithelium (Speare and Ferguson, 2006). Given this multifunctionality, compromised gills will not only affect respiration, but also impact other important physiological functions.

Today, a huge number of fish are used for experimental studies (Rønning, 2021). However, there is a drive towards reducing these fish numbers, and developing robust, biologically relevant, *in vitro* models for studies of infectious disease and toxicology (Paparella et al., 2021). In response to this, many fish cell lines have been developed. At the time of writing, Cellosaurus (version 45), the knowledge base of current cell lines used in biomedical research (Bairoch, 2018), listed 912 fish cell lines in its database, including 22 cell lines from Atlantic salmon. From the closely related salmonid, Rainbow trout (*Oncorhynchus mykiss L*), 77 cell lines were listed including the well-established and widely used gill epithelial cell line RTgill-W1 (Bols et al., 1994). Indeed, the validity and value of using fish cell lines was recently re-enforced with the establishment of an ISO standard (ISO21115) for water quality utilizing RTgill-W1 in a bioassay. This assay was also included in an OECD guideline in 2021 (OECD, 2021). Despite the increased number of fish cell lines developed in recent times, the development of a stable gill cell line from Atlantic salmon has remained elusive. In 2018, two gill cell lines from Atlantic salmon, named Atlantic salmon gill (ASG) −10 and ASG-13 were presented (Gjessing et al., 2018). This study suggested that ASG-10 cells are epithelial while ASG-13 cells are fibroblast-like. Both cell lines were observed to be susceptible to several viruses associated with severe diseases in Atlantic salmon. Our recent study (Solhaug et al., 2022) revealed differences between RTgill-W1 and ASG-10, where RTgill-W1 was far more sensitive to oxidative stress than ASG-10. Whether or not this *in-vitro* observation is a true reflection of fundamental differences between rainbow trout and Atlantic salmon gills *in-vivo*, remains to be elucidated.

To further characterize the ASG-10 gill cell line, and assess its value for future *in vitro* gill research, we have performed functional assays as well as transcriptomic and proteomic profiling, focusing on epithelial cell functions, such as barrier function, ion transport, phagocytosis, immune response, biotransformation and ABC transporters.

## 2 Materials and methods

### 2.1 Cell culture

The ASG-10 and ASG-13 cell lines were previously developed at the Norwegian Veterinary institute (Gjessing et al., 2018). The cells were grown in Leibowitz´s L-15 Glutamax (Gibco, Thermo Fisher, Waltan, MA, USA) medium supplemented with 10% FBS (USA origin, Gibco), 1% penicillin/streptomycin (Gibco) and 30 µM β-mercaptoethanol (Gibco), at 19°C in a non-ventilated cell culture flask and sub-cultured 1:2 every 10-day following detachment by using trypLE (Gibco). The temperature was chosen to optimize cell growth. For experiments, the cells were plated out at a concentration of 132 000/cm^2^ using complete cell culture medium without β-mercaptoethanol. The cell lines were routinely checked for *mycoplasma* infection using the MycoAlert^®^ *Mycoplasma* detection kit (Lonza, Basel, Switzerland).

### 2.2 Staining for mucous cells and mitochondria rich cells

To assess for the presence of mucous cells, the cells were seeded in 6-well plates and after 5 days the cells were fixed for 10 min in 4% paraformaldehyde at room temperature, washed with PBS, and stained with Periodic acid-Schiff (PAS), as described in (Suvarna et al., 2019). The cells were visualized with Leica DMIL microscope (Leica, Wetzlar, Germany), equipped with a color camera. For staining for mitochondria-rich cells (chloride cells), the cells were seeded on microscopy polymer coverslips (Ibidi, Gräfelfing) and after 5 days the mitochondria were stained with MitoTracker Red CMXRos (Molecular Probes, Invitrogen; 40 nM) in L-15 culture medium for 20 min at 19°C. The staining solution was replaced with phenol red free L-15 medium (Gibco) and mitochondria visualized by confocal microscopy (LSM710, 63x oil objective, Zeiss; Carl Zeiss Microscopy GmbH, Jena, Germany). For quantitative analyzing of differences in mitochondrial mass, the cells were harvested 5 days after plating (132 000/cm^2^, as described above), by using TrypLEe and stained with Mitotracker Green for flow cytometric analysis as described by the producer (Molecular probes, Invitrogen), and analyzed by flow cytometry (Accuri C6, BD Biosciences, Franklin Lakes, NJ, USA).

### 2.3 Phagocytic assays

The cells were plated out in 12-well plates (132 000/cm^2^) the day before the experiment. The cells were then exposed to pHrodo Red *E. coli* Bioparticles conjugate (Thermo Fisher, Invitrogen) 25 μg/ml, or pHrodo Red Zymosan A Bioparticles conjugate (Thermo Fisher, Invitrogen) 25 μg/ml in complete cell culture media. The particles were non-fluorescent and had neutral pH. As they are internalized through the phagocytic pathway, they became fluorescent as the pH of their surroundings decreased (within acidic lysosomes). After 48 h the cells were washed twice with PBS, counter stained with DAPI (1:1,000, Thermo Fisher, Invitrogen) and imaged using a fluorescent microscope (Echo Revolve microscope, Echo a BICO company, San Diego, CA, USA). For quantification, the cells were harvested by using trypLE, and fluorescence measured by flow cytometry (Accuri C6, BD, Franklin Lakes, NJ, USA).

### 2.4 Transepithelial electrical resistance (TER) assay

The cells were seeded on transwell inserts (Costar 0.4 µm polyester membrane; 132 000 cells/cm^2^) and TER was automatically measured using a CellZscope E (Nano Analytics, Münster, Germany).

### 2.5 Immunostaining for the junctional adapter protein ZO-1

The ASG-10 cells were seeded on transwell inserts (1.12 cm^2^, Costar 0.4 µm polyester membrane; 132 000 cells/cm^2^) and grown for 1–10 days. The cells were washed once in PBS (Gibco) and fixed with 4% paraformaldehyde (Sigma-Adrich, St-Louis, MO, USA) for 15 min at room temperature. The cells were then washed 3 times with PBS and blocked in 3% BSA (Sigma-Aldrich)/PBS for 60 min at room temperature. The membranes were then cut out from the insert using a scalpel (nr.12) and placed with the ASG-10 cells faced up in a 12-well plastic plate. The cells were then permeabilized using 0.05% saponin (Sigma-Aldrich)/3%BSA/PBS for 10 min and then incubated with a ZO-1 monoclonal antibody (ZO1-1A12, Thermo Fisher; 1:100) in 0.05% saponin/3%BSA/PBS buffer overnight at 4°C. The cells were then rinsed twice with PBS and incubated with a secondary antibody conjugated with Alexa Fluor 488 (AF488 PLUS a-mouse, 1:1,000, Molecular probes) and phalloidin 555 (1:400, Cell Signaling Tec, Danvers, MA, USA) for 1 h at room temperature. The cells were then washed 3 times with PBS, the nuclei stained with DAPI (1:1,000, Molecular probes) in the PBS wash (5 min). The membranes were then mounted between a microscope glass slide and a coverslip with ProLong Gold mounting medium (Thermo Fisher). Confocal fluorescence microscopy was performed using a Zeiss LSM 710 microscope and a ×40 oil objective lens. For ASG-10/ASG-13 co-culture: the ASG-13 cells were seeded on the basolateral side of the transwell inserts (132 000 cells/cm^2^) as followed: The inserts were placed in a 6-well plate with the basolateral side up. The cells were then seeded on the membrane in 200 μl cell culture medium. After 3 h in the incubator, the cells were attached to the membrane, and the inserts were then flipped and placed with the basolateral side down, and the ASG-10 cells were then seeded in the apical compartment and stained as described above. The immunostaining was done as described above.

### 2.6 Lucifer yellow permeability analysis

ASG-10 and ASG-10/ASG-13 co-culture were seeded on transwell inserts as described in 2.5 and grown for 10 days. TER was measured as described in 2.3 to ensure that the membranes were intact. The inserts were washed with HBSS (Lonza) three times and put into a new 12-well plate. The apical compartment was filled with 500 µl of Lucifer yellow (60 μM, Sigma Aldrich) dissolved in complete L-15 medium, and the basolateral compartment with 1,500 µl L-15 growth medium. The samples were then put on gentle agitation (50 rpm) for 2 h at room temperature. For control, a transwell insert without cells was used. The apical and basolateral medium were diluted 1:10 and the Lucifer yellow fluorescence measured using a plate reader (Spectramax i3x, Molecular devices, San Jose, CA, USA). Lucifer yellow permeability was calculated as described in Frost et al. (2019) and normalized to control.

### 2.7 ABC transporter assay

The cells were plated out in black 96-well plates (132 000/cm^2^) the day before the experiment. The cells were then pre-incubated in L-15 medium with different ABC transporter inhibitors: PSC833 (Sigma-Aldrich), MK751 (Sigma-Aldrich), Ceefourin (Bio-teche, MI, USA) and Probenecid (Biotinum, Fremont, CA, USA), for 15 min before the addition of Calcein-AM (0.25 µM; Sigma-Aldrich). Calcein-AM was dissolved in L-15 medium with the inhibitors, thus all incubations from this point onwards were carried out in the presence of inhibitors. After 1 h incubation the medium was removed, and the cells lysed with PBS/1% Triton. The cellular Calcein-AM uptake was then measured and quantified using a Spectramax i3x plate reader in fluorescence mode (Ex 494 nm/Em 517 nm). To ensure a representative readout of the fluorescent adherent cells, 37 different points were read in each well by using the well scan function of the plate reader. Caco-2 cells were used as a positive control, showing accumulation of Calcein-AM when using similar concentrations of PSC833 and MK751 as with the ASG-10 cells (data not shown).

### 2.8 Western blotting for P-glycoprotein (PGP) expression

The ASG-10 cells were seeded as described in 2.1. For positive control of PGP expression, we used Caco-2 cells. The cells were grown as described in (Ivanova et al., 2018) and confluent cells were used in the experiment. For cell lysis the ASG-10 and Caco-2 were washed twice in ice cold PBS and placed at −70°C until the next day. The cells were then scraped in cell lysis buffer (Cell Signaling Tec, Beverly, MA, USA) added 0.8% SDS and 1% proteinase inhibitor (sigma Aldrich). The samples were then centrifuged (10 000 x g, 1 min) through a QIAshredder (Qiagen, Hilden, Germany) for homogenization. The protein concentrations were quantified by using Bio-Rad DC protein assay kit (Bio-Rad Laboratories Inc., Hercules, CA, USA). Western blotting was performed by using the NuPage Novex system from Invitrogen. For detection of PGP the monoclonal antibody C219 (MA1-26528, Thermo Fisher; 1:180), shown to be reactive against Atlantic salmon (Tribble et al., 2008), was used as the primary antibody, and horseradish peroxidase-conjugated anti-mouse (1:500; Cell Signaling Tec) was used as the secondary antibody. For visualization, chemiluminescence, SuperSignal West Pico PLUS substrate (Thermo Scientific) and Chemidox XRS+ (BioRad, Hercules, CA, USA) were used.

### 2.9 Preparation of ASG-10 and gills for proteome and RNAseq analysis

A total of 5 Atlantic salmon parr (Fanad strain) aged 10 months were taken from freshwater tanks where they had been kept under low-light conditions. The median fish weight was 40.5 g (range: 33.4 g). The median fish length was 14.5 cm (range: 3.5 cm). The fish were euthanized with an overdose of Tricaine-methanesulfonate (Merck, Taufkirchen, Germany). Prior to gill excision, the gills were perfused by injection of sterile saline solution through the heart. All 8 gills from each fish were excised and the gill arch was removed using a sterile scalpel. Gills from the left side of the fish were stored in 1 mL of RNA later (Thermo Fisher Scientific, Waltham, MA USA) followed by storage at −80°C until required for further processing. Gills from the right side of the fish were flash frozen in liquid nitrogen, followed by storage at −80°C until required for further processing. ASG-10 cells were seeded in 75 cm^2^ cell culture flasks (132 000/cm^2^). Upon confluence, the cells were washed twice in ice-cold PBS and scraped with a cell scraper in 1 ml ice-cold PBS. The samples were centrifuged (600 x g, 4°C, 5 min) and the PBS removed. The samples were then snap frozen in liquid nitrogen and stored at −80°C until required for further processing.

### 2.10 Proteomic analysis

Total protein was extracted and prepared for LC-MS/MS analysis from samples from perfused juvenile salmon gills (n = 5), and from confluent ASG-10 cultures (n = 5). Sample preparation was carried out using the PreOmics iST kit (PreOmics, Martinsried, Germany) according to the Tissue/FFPE sample preparation protocol. Peptide fractions were analyzed on a quadrupole Orbitrap (Q-Exactive, Thermo Scientific) mass spectrometer equipped with a reversed-phase NanoLC UltiMate 3000 HPLC system (Thermo Scientific). Raw data from the Orbitrap Q-Exactive was processed using MaxQuant version 1.6.6.0 for identification of proteins (Cox and Mann, 2008). To identify peptides and proteins, MS/MS spectra were matched to the protein database of ENSEMBL release 99 (*Salmo salar*). Proteins were quantified using the LFQ algorithm in MaxQuant using default settings (Cox et al., 2014). It should be noted that a fold-change cut-off was not applied to the proteomic data.

### 2.11 Transcriptome analysis

The RNA used for RNA-seq analysis were prepared from the ASG-10 cells and gill samples described in Section 2.9. For harvesting ASG-10, the cells were washed twice with cold PBS, lysed in RNEasy lysis buffer (Qiagen, Hilden, Germany) and collected using a cell scraper. DNA was shredded by pipetting 5 times through a 20 G syringe until the sample was no longer viscous. Gill samples stored in frozen RNALater (Merck, Darmstadt Germany) were transferred to 0.5 ml Qiazol (Qiagen) in a 2 ml tube containing a 5 mm stainless steel bead (Qiagen), homogenized on a TissueLyser II (Qiagen) at 24.7 Hz for 2 × 5 min, added 0.1 ml chloroform (Merck), vortexed and centrifuged in a table centrifuge (10 min, 4°C, 10 000 x g), after which the aquatic top phase was collected for further isolation. RNA was isolated from gill and ASG-10 samples using a manual RNEasy total RNA kit (Qiagen) according to the manufacturer’s protocol. Eluted RNA samples from gills or cells were treated with Turbo DNase (Thermo Fisher scientific), and the resulting RNA was quantified on a Multiscan SkyHigh spectrometer (Thermo Fisher scientific), and quality checked on a 4200 TapeStation System (Agilent, Santa Clara, CA, USA) to ensure a RIN value above 8. Samples were then frozen at −80°C until the pure total RNA (1 µg) was sent to the Norwegian Sequencing Center (Oslo University Hospital, Norway) for library preparation and sequencing. Sequencing libraries were prepared using TruSeq mRNA library preparation kit (Illumina Inc., San Diego, CA, USA) following the manufacturer’s protocol. Twelve barcoded libraries were pooled together and sequenced on one lane of a NovaSeq SP flowcell (Illumina Inc.) to generate 150 bp paired-end reads. Raw data from the sequencer were cleaned using bbduk, BBMap toolkit v34.56 to remove/trim low-quality reads and adapter sequences (parameters: ktrim = r k = 23 mink = 11 hdist = 1 tbo tpe qtrim = r trimq = 15 maq = 15 minlen = 36 forcetrimright = 149). Cleaned reads were aligned against the *Salmo salar* ENSEMBL release 99 genome and annotation information using HiSat2 v2.1.0 (Kim et al., 2019) (parameters: --rna-strandness RF). Reads mapping to the known genes were calculated using FeatureCounts v1.4.6-p1 (parameters: -p -s 2) (Liao and Smythshi, 2013) and the differential expression analysis were carried out using DESeq2 v1.22.1 (Love and Huberanders, 2014) in R v3.5.1 using default settings. For further analysis, both transcriptomic and proteomic identifications were combined into one dataset and a cut-off based on a minimum detection of 10 reads was applied. It should be noted that a fold-change cut-off was not applied to the transcriptomic data. Gene ontology (GO) analysis was carried out using g:Profiler (Raudvere et al., 2019) using the *Salmo salar* reference dataset.

### 2.12 Statistical analysis

The data analyses for Figures 5, 6 were performed using Sigma Plot version 14.0. Statistical significance was considered at *p* < 0.05. The different tests used are specified in the figure legends. For bioinformatic analysis, LFQ values obtained in MaxQuant were imported into Perseus software v1.6.2.3 (Tyanova et al., 2016). Differentially expressed proteins (ASG-10 vs. gills, Table 1) were identified using a two-way Student’s t-test (*p* < 0.05) followed by Benjamin-Hochberg false discovery rate (FDR) correction. Differential expression analysis for the transcriptome data (ASG-10 vs. gill, Table 1) was carried out using DESeq2 v1.22.1 (Love and Huberanders, 2014) in R v3.5.1 using default settings.

**TABLE 1:** Summary of proteomic and transcriptomic identifications (gene and protein ID count). *p*-value cut-off for gene expression analysis < or = 0.05; *p*-value cut off for protein analysis < or = 0.05. A cut-off based on a minimum of 10 reads was applied to the transcriptomic data. Log 2-fold change (Log2fc) for proteins with significant differences: −0.3 to −4.6 (lower); 0.5 to 5.0 (higher). Log2fc for genes expressed with significant differences: −0.3 to −12.7 (lower); 0.2 to 9.0 (higher).

*Gene name*	*Gene/Protein ID counts*	*% of total*
Proteins with no significant differences	1,406	59
Proteins lower in ASG-10	931	39
Proteins higher in ASG-10	39	2
		
Total proteins	2,376	
		
Genes expressed with no significant differences	6,044	26
Genes expressed lower in ASG-10	7,696	34
Genes expressed higher in ASG-10	8,986	40
Total number of genes expressed in both ASG-10 and gill	22726	
		
Genes expressed in ASG-10 only	1849	-
Genes expressed in gill only	7,057	-
Total genes expressed overall	31632	

## 3 Results

### 3.1 Biophysical characterization

#### 3.1.1 Cell homogeneity

**FIGURE 1:**
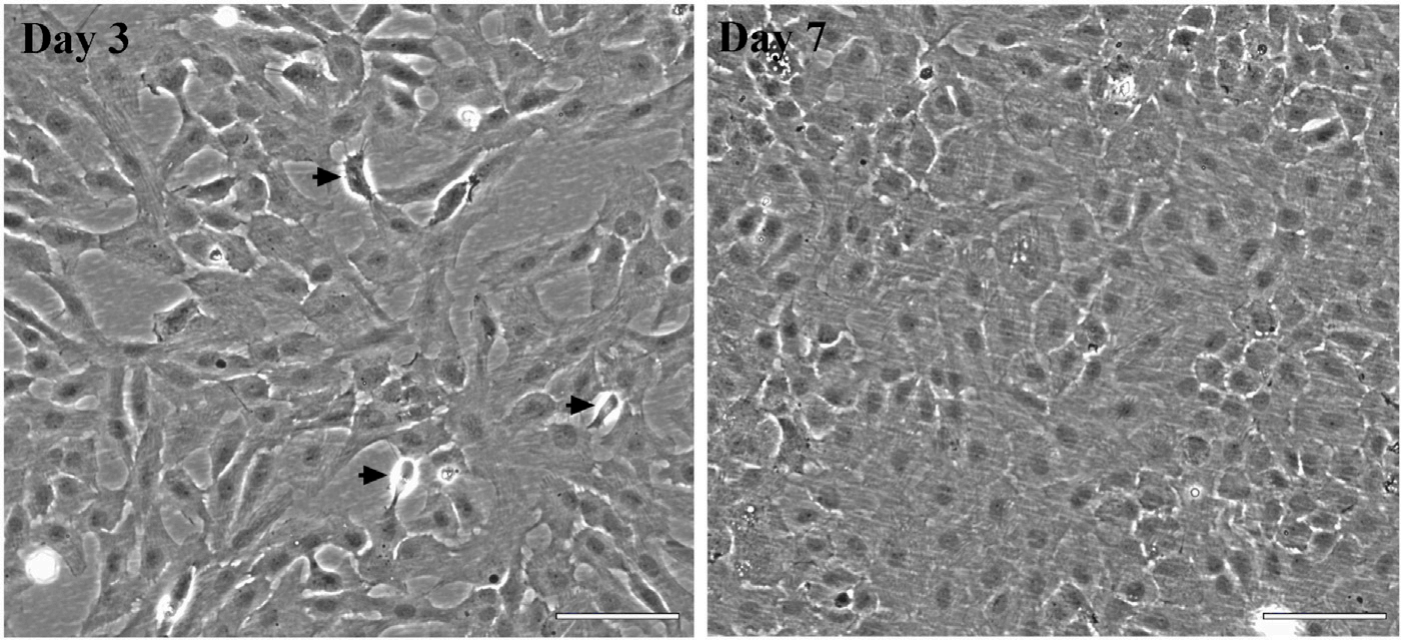
Morphology of the ASG-10 cell line. Phase contrast micrographs of representative cultures of ASG-10 cells at day 3 (proliferating) and 7 (confluent) after seeding. Arrows indicate mitotic cells. The images are taken with a phase contrast microscope (Zeiss Observer A1).Scale bar = 100 µm.

**FIGURE 2:**
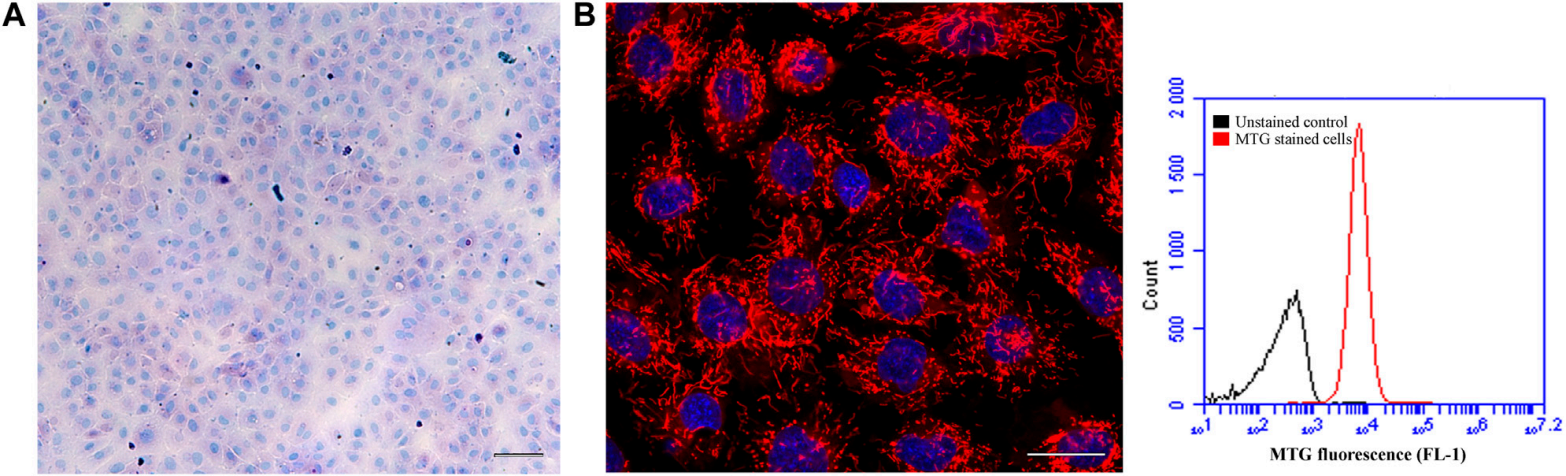
The ASG-10 cell line seems to contain neither goblet nor chloride cells. The ASG-10 cells were grown for 5 days. **(A)** The cells were stained with PAS to identify goblet cells (bright pink) and visualized by bright field microscopy. Scale bar = 100 µm. **(B)** The cells were stained with mitotracker red to identify cells rich in mitochondria and visualized by confocal fluorescence microscopy. Red = mitochondria, Blue = nuclei. Scale bar = 20 µm. For quantification of mitochondrial mass, the cells were stained with mitotracker green (MTG) and analyzed with flow cytometry. Flow cytometric histograms showing relative amount of mitochondrial mass in each cell are shown.

Figure 1 shows the ASG-10 cells in proliferative and confluent state visualized by phase contrast microscopy. We examined the homogeneity of the cell line by staining the cell population for goblet and chloride cells 5 days after plating. A positive PAS staining would appear as bright pink staining of the whole cell or just the mucous granules in the cell (Smith et al., 2018). In the absence of a mucous cell line from fish and the differences in the methodology in staining of gill tissue and a cell line, a good positive staining control is absent. However, none of the stained ASG-10 cells appears to contain bright pink granuoles (Figure 2A). The seemingly negative PAS staining suggested that no goblet cells were present in the ASG-10 cell culture. Further investigation of the presence of goblet cells, expression of mucins, in the cell culture were done with transcriptomic analysis (Section 3.2.3). To examine the presence of chloride cells, which are specialized mitochondria rich epithelial cells, we used mitotracker red and confocal microscopy to examine the mitochondria in detail. None of the cells appeared to have more mitochondria compared to other cells in the culture (Figure 2B). To investigate further, we quantified mitochondrial mass by staining the cells with mitotracker green and analyzed by flow cytometry. In agreement with the confocal images, only cells with approximately similar mitochondrial mass were detected (Figure 2B). Correspondingly, when examining TEM data from our previous study (Gjessing et al., 2018), no mitochondria-rich cells were observed (data not shown). Thus, the ASG-10 cell line appears to contain neither goblet nor chloride cells.

#### 3.1.2 Phagocytosis

**FIGURE 3:**
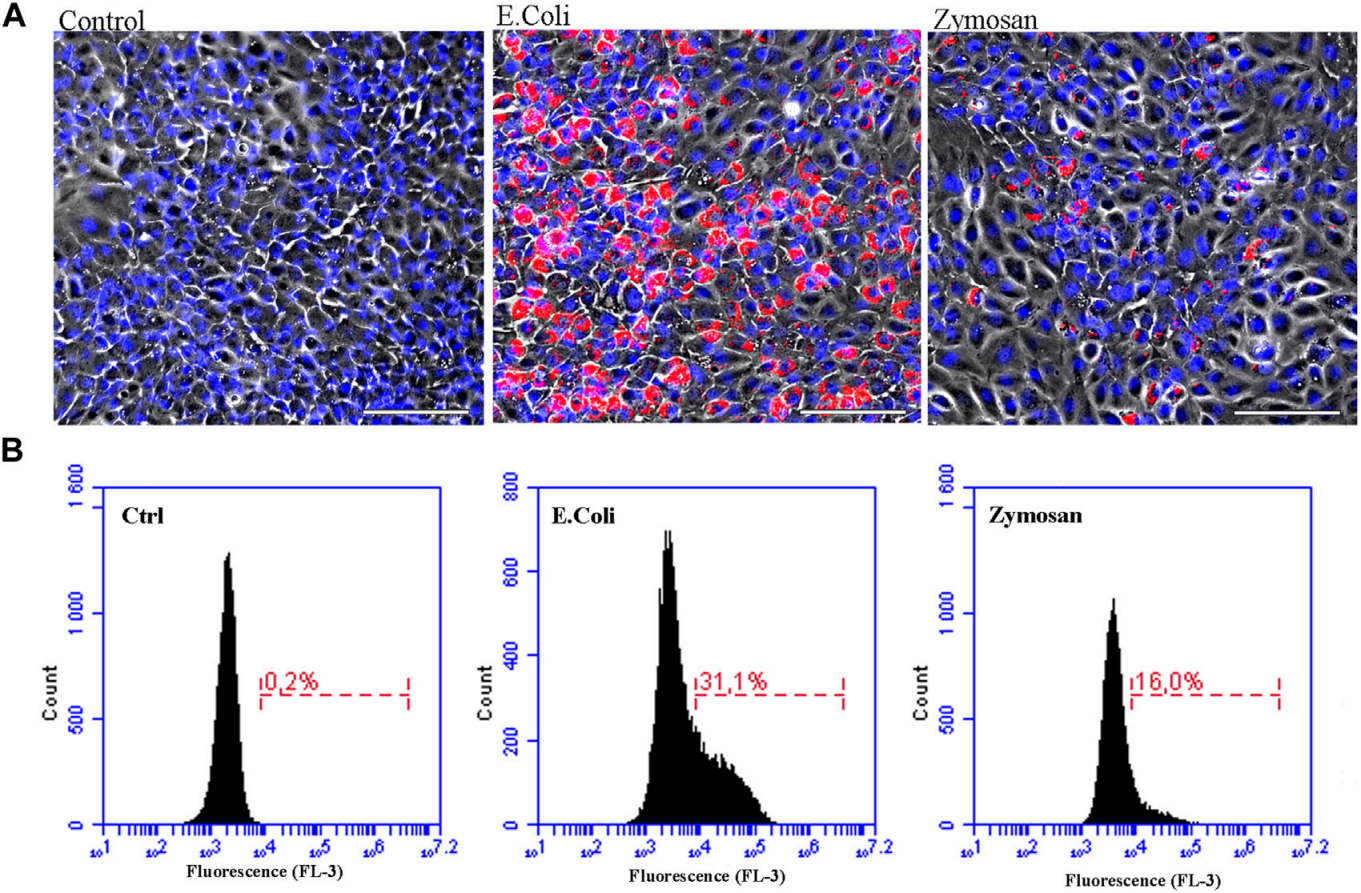
The ASG-10 cell line has phagocytic activity. ASG-10 was exposed to pHrodo Red *E. Coli* or Zymosan A bioparticles (50 μg/ml) for 48 h and analyzed for phagocytic activity by **(A)** fluorescence microscopy (scale bar = 100 μm, Blue = nuclei, Red = pHrodo particles taken up by the cells) and **(B)** flow cytometry. Percentage of cells that have taken up particles (red fluorescent cells) are shown.

To investigate the phagocytic activity of the ASG-10 cells, we used two different pHrodo Bioparticles, Zymosan A from yeast cell wall and the gram-negative bacteria *Escherichia coli (E. coli)*, which upon entering the acidic lysosomes become fluorescent. ASG-10 cells were able to take up both particles but *E.coli* were phagocytosed more efficiently than Zymosan A (Figure 3). This clearly shows that ASG-10 can phagocytize bioparticles, and there is a difference in the uptake of the yeast Zymosan compared to bacteria, which may be due to the involvement of different receptors in the process of phagocytosis.

#### 3.1.3 Barrier function

**FIGURE 4:**
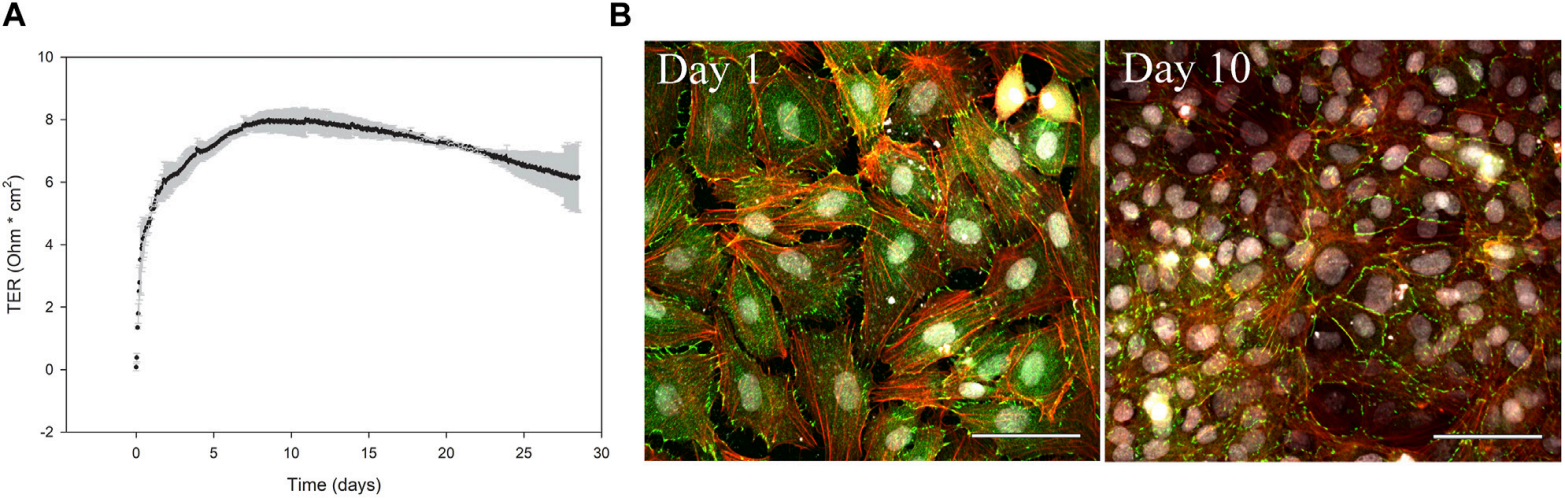
ASG-10 generates TER and express the junctional adapter protein ZO-1. ASG-10 was grown on transwell inserts for 28 days. **(A)** TER was measured every 2 h. Mean ± SD of 4 independent experiments are shown. **(B)** ASG-10 stained for ZO-1 (green), f-actin (red) and nuclei (grey) after 1 and 10 days following plating and visualized by confocal microscopy.

**FIGURE 5:**
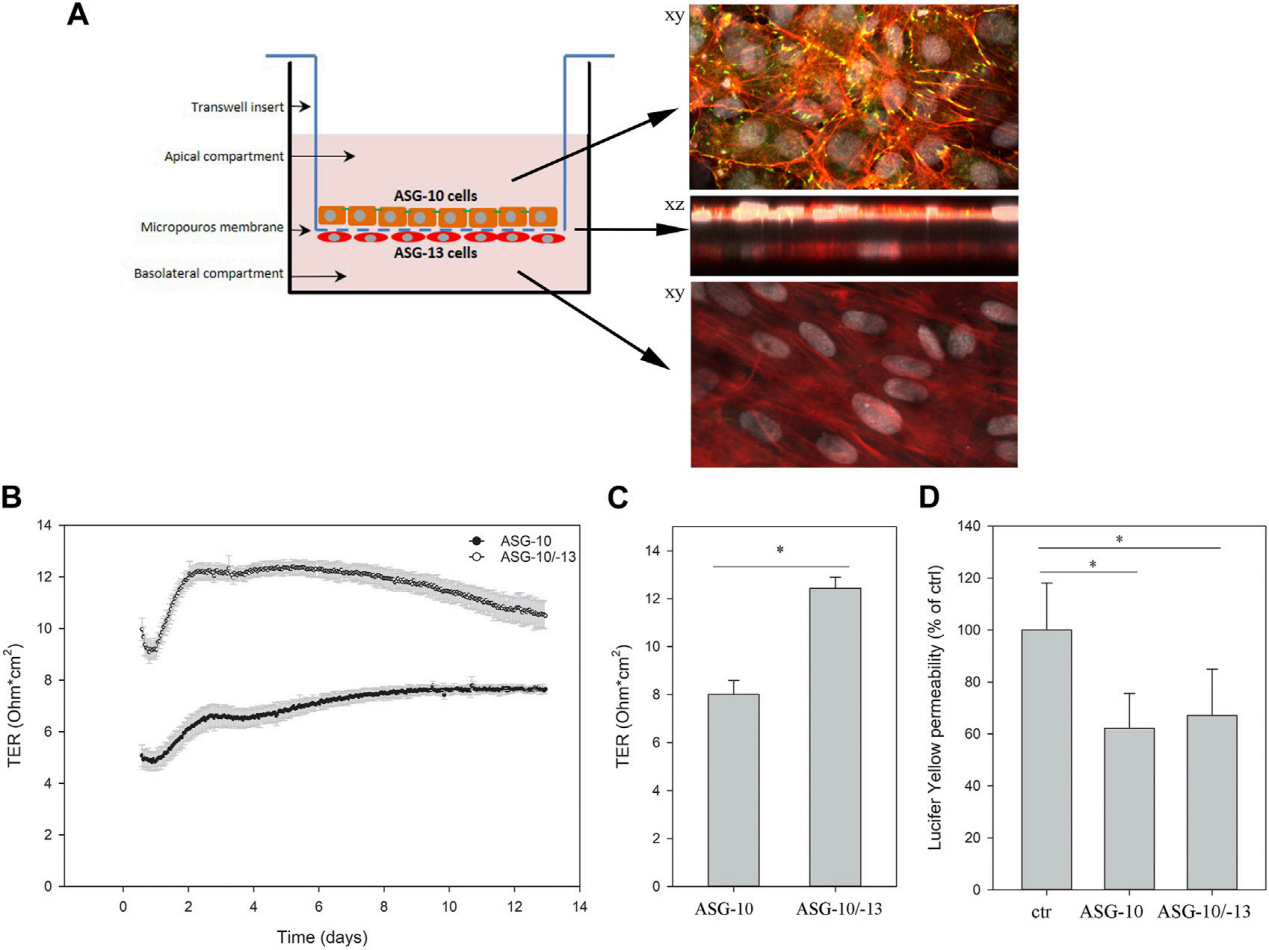
ASG-10 grown in co-culture with ASG-13 increased the TER, but had no impact on permeability of Lucifer yellow. ASG-10 was grown in the apical compartment and ASG-13 in the basolateral compartment for 7–14 days. **(A)** The ASG-10/ASG-13 co-culture were stained for ZO-1 (yellow-green), phalloidin (red) and nuclei (grey) after 10 days in culture and visualized by confocal microscopy. **(B)** TER was measured every 2 h for 13 days. Mean of three replicates +/- SD are shown. **(C)** Graph with statistical data are shown for day 7 and represent mean ± SD of 3 independent experiments. Statistical significance between the groups (*p* < 0.05, indicated by *) was assessed using paired *t*-test. **(D)** Permeability of Lucifer yellow. The data represent mean ± SD of three - five independent experiments. Statistical significance between the groups (*p* < 0.05, indicated by *) was assessed using 1-way ANOVA, mixed-effects analysis and Holm-Sidaks post-test.

The formation of a selectively permeable barrier is an important functional characteristic of epithelial cells. Transepithelial electrical resistance (TER) is a widely accepted quantitative measurement of the integrity of a cellular monolayer and tight junction dynamics (Srinivasan et al., 2015). To evaluate the ability of the ASG-10 cells to generate TER, the cells were seeded on transwell membranes, and TER was measured every 2 h for 28 days. During the first 5 days the TER increased and gradually reached a peak after about 8–10 days at approximately 8 Ω * cm^2^. Then, TER gradually decreased to about 6 Ω * cm^2^ after 28 days (Figure 4A). Tight junctions are important for the generation of a selective barrier. In the ASG-10 cells, the junctional adapter protein ZO-1 was clearly expressed. At day 1 after plating, ZO-1 staining could be seen both in the cytoplasm and at the outer edges of the cells, while at day 10 it was mainly expressed at cell-cell junctions (Figure 4B), resembling a tight barrier. Thus, the generated TER corresponded to ZO-1 expression and to the increased cell number. Using more complex cell models often enhances the accuracy of extrapolation to the *in vivo* situation. We therefore made a co-culture of ASG-10 and the Atlantic salmon gill fibroblast-like cell line ASG-13. Here ASG-10 was grown apically and the ASG-13 basolaterally on a transwell membrane (Figure 5A). The ASG-10/ASG-13 co-culture made a confluent layer on each side of the membrane and co-existed well under similar growth conditions. Together the cells generated significantly higher TER compared to ASG-10 alone (Figures 5B, C). The permeability test performed by monitoring the leakage of Lucifer yellow between the compartments showed significant differences from control but there was no difference between ASG-10 alone or the co-culture with ASG-13 (Figure 5D).

#### 3.1.4 ABC transporters

**FIGURE 6:**
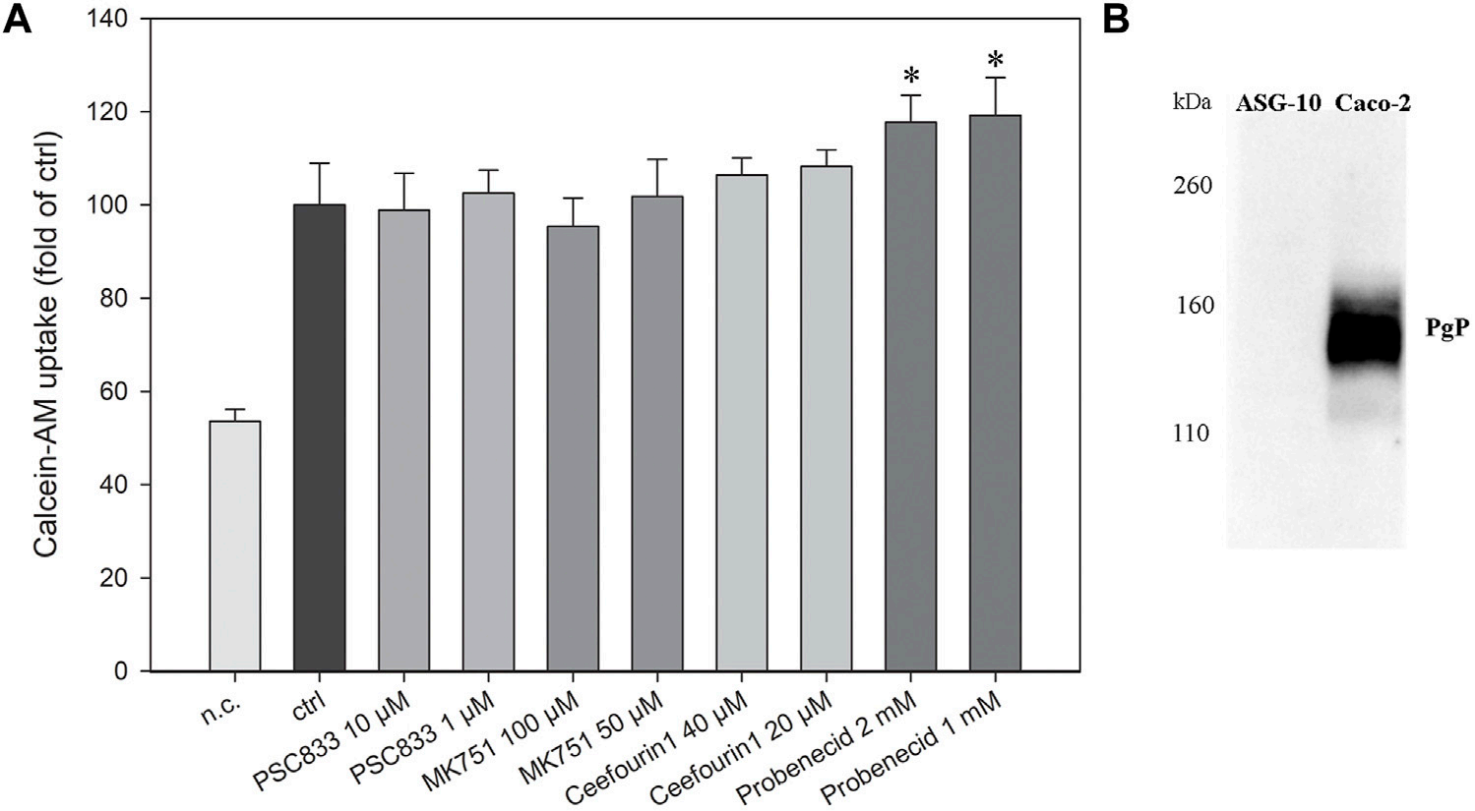
Characterization of efflux transport proteins in ASG-10 by using specific inhibitors and the Calcein-AM transport assay. **(A)** The ASG-10 cells were treated with different efflux transport protein inhibitors (PCS833: PGP/MDR1/ABCB1-inhibitor, MK751: MRP1/4-inhibitor, Ceefourin1: MRP4-inhibitor, Probenecid: MRP/organic-anion transporter inhibitor) and transport during 1 h measured by the Calcein-AM transport assay. n.c= negative control. The data represents mean ± SD of four independent experiments. Statistical significance between the groups (*p* < 0.05, indicated by *) was assessed using 1-way ANOVA with Holm-Sidaks post-test. **(B)** Pgp (141 kDa) expression in ASG-10 and Caco-2 (positive control) measured by western blot.

The current knowledge of the presence of ABC transporters in gills is sparse, however a recent study on Rainbow trout suggested functional ABC transporter activity in the gills (Kropf et al., 2020). Therefore, we wanted to investigate ABC transporter activity in the ASG-10 cell line using an ABC transport assay. Calcein-AM is a cell permeable dye and a substrate for several efflux transporters. It easily diffuses into the cells, where the ester bond is cleaved by esterases to calcein, which is fluorescent. Several transporters extrude Calcein-AM from the plasma membrane. However, when these transporters are inhibited, more Calcein-AM enters the cells. Transporter activity is therefore indicated by an increase in the accumulation of Calcein-AM in the cells in the presence of an inhibitor. Several known transporter inhibitors were tested, namely PSC833 (PGP/multidrug resistance protein-1 (MDR1)/ABCB1 inhibitor), (Tai, 2000), MK751 (multidrug resistance-associated protein 1 and 4 (MRP1/4), (Hofmann et al., 1995; Chen and Leekruh., 2001; Reid et al., 2003), ceefourin1 (MRP4 inhibitor), (Cheung et al., 2014), and probenecid (MRP/organic-anion transporter inhibitor) (Gollapudi et al., 1997). Interestingly, only probenecid showed a significant increase in accumulation of Calcein-AM (Figure 6A). Furthermore, the lack of PGP protein expression in the ASG-10 cells, as determined by western blotting using the C219 monoclonal antibody (Figure 6B), corresponds to the unresponsiveness of the PCS833 inhibitor in the Calcein-AM assay.

### 3.2 Proteomic and transcriptomic analysis

#### 3.2.1 Summary of proteomic and transcriptomic data

The total number of protein identifications from the initial MaxQuant mass spectrophotometric data output was 4,112. Following preliminary processing, the total number of proteins detected and compared in both ASG-10 and gill was 2,376. The majority of these proteins (1,406; 59%) were present at similar levels in the cell line and gill (*p* > 0.05; Table 1). Thirty-nine proteins (2%) were detected at a higher level in ASG-10 cells relative to gill, with 931 proteins detected at a lower level (39%; *p* < 0.05).

RNA-seq produced 42 million 150 bp pair-end reads on average for each sample. More than 98% of reads survived pre-processing and the resulting 90% of the clean reads aligned to the Atlantic salmon genome (Supplementary Table S1). The total number of genes detected and compared in both ASG-10 and gill was 22,726. A smaller proportion of genes were detected at a similar level in cells and gill (6,044; 26%; *p* > 0.05). A total of 8,986 genes (40%) were detected at a higher level in ASG-10 cells, compared with 7,696 detected at a lower level (34%; *p* < 0.05). The total number of genes detected in ASG-10 only was 1849. The total number of genes detected in gill only and not in ASG-10 was 7,057.

#### 3.2.2 Gene ontology (GO) analysis

To gain greater insight into the similarities and differences between ASG-10 and gill, GO analysis was performed using g:Profiler. Separate analyses were carried out for both the transcriptomic and proteomic datasets (using corresponding gene ids). The top 10 molecular functions and biological process terms that emerged from the analysis of each dataset are presented in Supplementary Figure S1.

**FIGURE 7:**
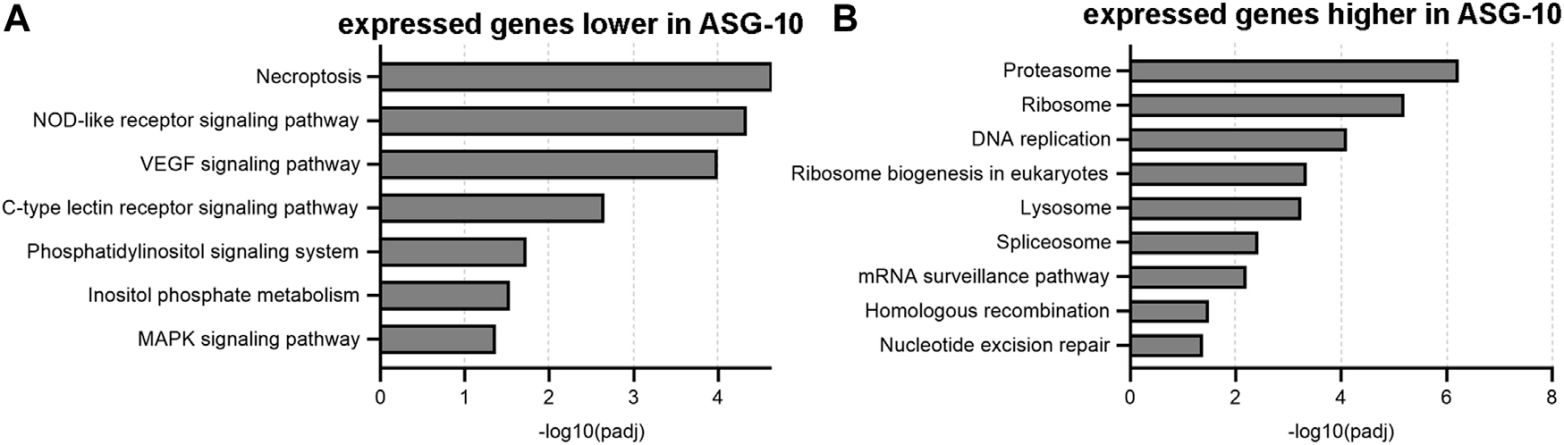
Summary of KEGG terms ranked according to the enrichment score (-log10padj value). **(A)** KEGG terms emerging from 7,696 genes expressed at significantly lower levels in ASG-10. **(B)** KEGG terms emerging from 8,986 genes expressed at significantly higher levels in ASG-10. The higher the enrichment score value, the more significant the pathway is for the given set of genes.

The top 10 KEGG terms for the transcriptome data comparison between gills and ASG-10 cells, presented as a function of decreasing enrichment score (represented as–log10[padj]), are displayed in Figure 7, with additional comparisons included in Supplementary Figure S2. The KEGG term with the greatest enrichment score for the gene expression detected at a lower level in ASG-10 was necroptosis, which relates to cell death (Figure 7A). In addition, multiple cell signaling pathway terms, including those associated with immune and inflammatory responses, such as the NOD-like receptor, C-type lectin receptor and MAPK signaling pathway, were associated with lower detection levels (Figure 7A).The most significant terms associated with higher detection levels in ASG-10 compared with gill were those associated with the proteasome (proteolysis), the ribosome (translation), the spliceosome (RNA splicing) and DNA replication (Figure 7B). At the lower enrichment score level, DNA repair and surveillance were evident.

The proteomic data showed differences in proteins involved in housekeeping functions like metabolic pathways and protein synthesis and degradation (Supplementary Figure S2). The number of proteins detected at a higher level in ASG-10 compared with gill was limited (39 proteins). Twelve proteins were detected with a fold change of greater than or equal to 1.5. Of these, 4 were components of respiratory chain proteins including NADH:ubiquinone oxidoreductase subunit B1, cytochrome b-c1 complex subunit Rieske mitochondrial-like, NADH dehydrogenase and cytochrome c. Two were involved in actin severing, namely scinderin like b and cofilin-2-like. Other proteins within this group included a POU domain-containing transcription factor, a translation machinery protein and splicing factor proteins.

Expression of genes associated with the Fanconi anemia pathway, known to control repair of damaged DNA (Peake and Noguchi, 2022), appeared to be consistent in both the cell line and gill (Supplementary Figure S2). It appeared that expression detected only in ASG-10 involved biological transport functions, particularly ion transport. Cellular adhesion function also emerged. In line with this, molecular functions related to cation transporter activity, channel activity and passive transmembrane transporter activity were evident in ASG-10 (Supplementary Figure S1).

#### 3.2.3 Ion transporters and mucins

The ASG-10 cell line does not seem to contain mitochondrion-rich chloride cells (Figure 2B), but ion transporters of the Na^+^/H^+^ exchanger (NHE), solute carrier (SLC), NaCl co-transporter (NCC), Na^+^/K^+^-ATPase (NKK), or V-type ATPase (V) families may also be present in pavement epithelial cells, so the transcriptomic and proteomic datasets were interrogated to focus on ion transporters (Table 2). Expression of several Cl/HCO_3_^−^ anion exchanger genes from the SLC4a and SLC26a families were detected in ASG-10, most at a similar or higher level in the cells compared with gill. Transcription of 3 members of the SLC4a family were found in ASG-10 only. Similarly, the majority of NHE genes were expressed at similar or higher levels in ASG-10 compared with gill, with transcription of only one NHE-3-like gene detected in the cells only. No NCC transcripts or proteins were seen in ASG-10. Expression of 3 NKK α-subunits (α1, α3 and α3-like) along with 3 of the β subunits (β-3-like, β-233 and β-233-like) were detected, albeit at a lower level in ASG-10 compared with gill. The only exception was the β-3-like, which were detected at a significantly higher level in the cells. Expression of some genes of the V-type ATPase were detected. These genes encoded the membrane-associated V0 subunits (a, c, d and e) and membrane-embedded V1 subunits (A, C, D, E and F). The V1, E1-like and F protein subunits of the ATPase were also detected and were present at a similar level in both cells and gill.

**TABLE 2:** Profile of ion regulatory gene expression and proteins detected in ASG-10 relative to gill. Log2fc for protein with significant differences: −0.3 to −4.6 (lower); 0.5 to 5.0 (higher). Log2fc for genes expressed with significant differences: −0.3 to −12.7 (lower); 0.2 to 9.0 (higher).

*Gene name*	*Gene description*	*Gene ID*	*Gene Log2fc*	*Protein ID*	*Protein Log2fc*
Cl/HCO^3-^ exchanger					
slc4a2a	anion exchange protein 2-like	ENSSSAG00000048847	NA*	NA	NA*
slc4a3	anion exchange protein 3-like	ENSSSAG00000048003	NA*	NA	NA*
slc4a3	anion carrier family 4 member 3	ENSSSAG00000038490	NA*	NA	NA*
slc4a4b	solute carrier family 4 member 4b	ENSSSAG00000001818	−6.610	NA	ND
slc26a11	sodium-independent sulfate anion transporter-like	ENSSSAG00000008489	−3.424	NA	NA
slc26a9	solute carrier family 26 member 9-like	ENSSSAG00000074870	0.682	NA	ND
slc26a2	solute carrier family 26 member 2	ENSSSAG00000004653	1.090	NA	ND
slc26a5	prestin like	ENSSSAG00000003436	1.217	NA	ND
slc4a1ap	solute carrier family 4 member 1 adaptor protein	ENSSSAG00000009333	1.726	NA	ND
slc4a2b	anion exchange protein 2-like	ENSSSAG00000055848	1.941	NA	ND
Slc4a2b	anion exchange protein 2-like	ENSSSAG00000044249	2.400	NA	ND
slc26a6	solute carrier family 26 member 6-like	ENSSSAG00000067253	3.100	NA	ND
Na/H Exchanger					
slc9a3*	Na^+^/H^+^ exchanger 3-like	ENSSSAG00000055297	NA*	NA	ND
	Na^+^/H^+^ exchanger 3-like	ENSSSAG00000055927	−5.945	ENSSSAP00000065038	NS
slc9a6b	Na^+^/H^+^ exchanger 6b	ENSSSAG00000042506	−1.886	NA	ND
slc9a8	Na^+^/H^+^ exchanger 8	ENSSSAG00000009646	−0.914	NA	ND
slc9a1b	Na^+^/H^+^ exchanger 1	ENSSSAG00000017046	0.027	NA	ND
slc9a1	Na^+^/H^+^ exchanger 1	ENSSSAG00000073484	0.999	NA	ND
slc9a6a	Na^+^/H^+^ exchanger 6a	ENSSSAG00000076931	1.129	NA	ND
slc9a6a	Na^+^/H^+^ exchanger 6a	ENSSSAG00000063494	1.211	NA	ND
V-type ATPase					
	V1 ATPase subunit S1	ENSSSAG00000000842	NA*	NA	ND
atp6v0a1	V0 ATPase subunit a1	ENSSSAG00000056160	NA*	NA	ND
atp6v0a2a	V0 ATPase 116 kDa subunit a isoform 2-like	ENSSSAG00000011563	NA*	NA	ND
atp6v1c1a	V1 ATPase subunit C1a	ENSSSAG00000005321	NA*	NA	ND
atp6v0a1	V0 ATPase 116 kDa subunit a isoform 1-like	ENSSSAG00000009990	−1.896	NA	ND
atp6v0a2b	V0 ATPase 116 kDa subunit a isoform 1-like	ENSSSAG00000054796	−1.620	NA	ND
atp6v01e1	V0 ATPase e 1-like	ENSSSAG00000079359	−0.913	NA	ND
atp6v0a2a	V0 ATPase 116 kDa subunit a isoform 1-like	ENSSSAG00000050674	−0.518	NA	ND
atp6v0a1a	V0 ATPase 116 kDa subunit a isoform 1-like	ENSSSAG00000003758	NS	NA	ND
atp6v1e1a	V1 ATPase subunit E1-like	ENSSSAG00000010140	NS	ENSSSAP00000021002	
atp6v0d1	V0 ATPase d1	ENSSSAG00000041144	0.960	NA	ND
atp6v0ca	V0 ATPase ca	ENSSSAG00000053117	1.171	NA	ND
atp6v1a	V1 ATPase subunit A-like	ENSSSAG00000004368	1.385	NA	ND
atp6v1d	V1 ATPase subunit D-like	ENSSSAG00000043385	1.446	NA	ND
atp6v1f	V1 ATPase subunit F	ENSSSAG00000070564	1.825	ENSSSAP00000089523	
atp6v1c1	V1 ATPase subunit C1	ENSSSAG00000043463	1.843	NA	ND
atp6v1f	V1 ATPase subunit F	ENSSSAG00000056651	1.896	NA	ND
Na/K ATPase (NKK)					
	NKK subunit alpha 3-like	ENSSSAG00000043774	−5.110	NA	ND
atp1a3a	NKK subunit alpha 3	ENSSSAG00000071358	−2.874	NA	ND
	NKK subunit alpha 1	ENSSSAG00000041014	−2.612	ENSSSAP00000036495	−1.00
at233	NKK subunit beta-233	ENSSSAG00000006582	−1.466	ENSSSAP00000013183	NS
atp1a1	NKK subunit alpha 1	ENSSSAG00000007457	−1.191	ENSSSAP00000049677	−0.7
	NKK subunit alpha 3-like	ENSSSAG00000054749	−0.684	ENSSSAP00000102973	NS
at233	NKK subunit beta-233-like	ENSSSAG00000053842	−1.133	ENSSSAP00000055862	NS
	NKK subunit alpha 3-like	ENSSSAG00000074572	1.581	NA	ND
atp1b3b	NKK subunit alpha 3-like	ENSSSAG00000004867	3.538	NA	ND

Red, ASG-10 lower than gill; green, ASG-10 higher than gill; NS in grey, unchanged; NA* denotes gene expression detected in ASG-10 only. Log2fc, Log 2-fold change; ND, not detected; NA, not applicable.

Several mucin-like genes were expressed in ASG-10 (Table 3). The majority of these were expressed at a lower level in the cells compared with gill. Some mucin genes, however, were expressed only in ASG-10, including one mucin-2-like, a mucin 5AC like and a mucin 13-like. A mucin-17 like gene was expressed at a moderately higher level in ASG-10. It should be noted that no mucin proteins were detected in the proteomic data set.

**TABLE 3:** Profile of mucin gene expression detected in ASG-10 relative to gill. Log2fc for genes expressed with significant differences: −0.3 to −12.7 (lower); 0.2 to 9.0 (higher).

*Gene names*	*Gene description*	*Gene id*	*Log2fc*
	mucin-2-like	ENSSSAG00000081405	NA*
	mucin-5AC-like	ENSSSAG00000077246	NA*
muc13b	mucin-13-like	ENSSSAG00000069082	NA*
	mucin-17-like	ENSSSAG00000047299	NS
	mucin-2-like	ENSSSAG00000047682	−2.893
si:ch211-105j21.9	mucin-5AC-like	ENSSSAG00000051497	−5.35
	mucin-5AC-like	ENSSSAG00000077386	−0.485
mcama	mucin-18-like	ENSSSAG00000064621	−4.431

Red, ASG-10 lower than gill; green, ASG-10 higher than gill; NS in grey, unchanged; NA* denotes gene expression detected in ASG-10 only. Log2fc, Log 2-fold change; ND, not detected; NA, not applicable.

#### 3.2.4 Phagocytosis

The ASG-10 cells were more efficient in phagocytosis of *E.coli* compared to Zymozan A (Figure 3), which led us to investigate phagocytic receptors and opsonins. Clathrin heavy and light chain genes were expressed at a similar or higher level in cells compared with gill as were several dynamin 1 and 2 genes. The corresponding protein subunits were detected at slightly lower levels in the cells compared with gill. mRNA expression of several mannose binding lectins (mannose binding lectin 1, mannose binding lectin 2-like) was detected at higher levels in ASG-10 compared to gill while transcription of the mannan binding lectin serine peptidase 2 was detected in ASG-10 only (Table 4). The levels of many C-type lectins, galectins, fucolectins and sialic acid binding Ig-like lectins varied in the cells compared with gill, with some variants higher in ASG-10 and others higher in gills. Interestingly, high levels of transcription of a fucolectin 6-like gene were detected in ASG-10 only, along with CLEC 19A and some other lectins belonging to the mannan binding lectin, galectin and collectin families. Several C-type lectin domain family members were either absent from ASG-10 or dominated in gills (Table 4).

**TABLE 4:** Profile of phagocytic gene expression and proteins detected in ASG-10 relative to gill. Log2fc for genes expressed with significant differences: −0.3 to −12.7 (lower); 0.2 to 9.0 (higher).

*Gene name*	*Gene description*	*Gene ID*	*Gene Log2fc*	*Protein ID*	*Protein Log2fc*
Clathrin					
	Clathrin heavy chain 1	ENSSSAG00000000892	NS	ENSSSAP00000003061	−0.729
	clathrin heavy chain 1-like	ENSSSAG00000064444	NS	ENSSSAP00000086219	−3.124
clcb	*Salmo salar* Clathrin light chain B (clcb), mRNA.	ENSSSAG00000039138	NS	ENSSSAP00000030134	NS
	clathrin light chain A-like	ENSSSAG00000002775	1.86	NA	ND
	clathrin heavy chain 1-like	ENSSSAG00000078122	0.572	NA	ND
	clathrin heavy chain 1-like	ENSSSAG00000065798	1.045	NA	ND
	clathrin heavy chain 1-like	ENSSSAG00000079627	2.446	ENSSSAP00000116638	−0.704
cltcl1	clathrin heavy chain 1-like	ENSSSAG00000042487	2.592	NA	ND
Dynamin					
dnm1a	dynamin 1a	ENSSSAG00000054415	4.75	NA	ND
dnm2a	dynamin 2a	ENSSSAG00000000660	−1.085	ENSSSAP00000001549	−0.866
dnm3a	dynamin-2-like	ENSSSAG00000054999	−0.865	NA	ND
opa1	dynamin-like 120 kDa protein, mitochondrial	ENSSSAG00000044915	−0.049	NA	ND
	dynamin-1-like protein	ENSSSAG00000071783	1.242	ENSSSAP00000093032	−1.255
dnmbp	dynamin binding protein	ENSSSAG00000065884	1.385	NA	ND
DNM1L	dynamin-1-like protein	ENSSSAG00000007989	1.747	NA	ND
Lectins					
clec14a	C-type lectin domain containing 14A	ENSSSAG00000077789	−4.816	ND	ND
CLC4E	C-type lectin domain family 4 member E-like	ENSSSAG00000002892	−2.634	NA	ND
C209E	*Salmo salar* C type lectin receptor A (LOC100136446), mRNA.	ENSSSAG00000076658	−5.177	NA	ND
zgc:174904	*Salmo salar* C type lectin receptor C (LOC100136448), mRNA.	ENSSSAG00000070511	NS	NA	ND
lman1	lectin, mannose binding 1	ENSSSAG00000039268	0.571	NA	ND
lman2l	lectin, mannose binding 2 like	ENSSSAG00000046353	0.313	NA	ND
masp1	mannan binding lectin serine peptidase 1	ENSSSAG00000043562	−1.186	NA	ND
lgals3a	lectin, galactoside binding soluble 3a	ENSSSAG00000057680	1.626	NA	ND
leg	*Salmo salar* Beta-galactoside-binding lectin (leg), mRNA.	ENSSSAG00000000358	5.402	ENSSSAP00000000566	−0.565
lgals8	*Salmo salar* galectin 8 (lgals8), mRNA.	ENSSSAG00000075838	0.766	NA	ND
leg3	*Salmo salar* Galectin-3 (leg3), mRNA.	ENSSSAG00000071455	2.106	ENSSSAP00000091810	−0.578
lg3bp	*Salmo salar* Galectin-3-binding protein (lg3bp), mRNA.	ENSSSAG00000037936	−2.511	NA	ND
LEG2	galectin-2-like	ENSSSAG00000001248	2.026	NA	ND
	galectin-3-binding protein A-like	ENSSSAG00000075036	−2.649	NA	ND
lgals3a	galectin-3-like	ENSSSAG00000010091	2.916	NA	ND
LEG9	galectin-9B-like	ENSSSAG00000070750	2.925	ENSSSAP00000090053	NS
LEG9	galectin-9-like	ENSSSAG00000075631	NS	NA	ND
si:ch211-79k12.1	sialic acid-binding Ig-like lectin 16	ENSSSAG00000010668	−7.712	NA	ND
	collectin-12-like	ENSSSAG00000070680	−4.577	NA	ND
masp2	mannan binding lectin serine peptidase 2	ENSSSAG00000079450	NA*	NA	ND
lgalslb	lectin, galactoside-binding-like b	ENSSSAG00000053944	NA*	NA	ND
fel	*Salmo salar* Fish-egg lectin (fel), mRNA.	ENSSSAG00000009266	NA*	ENSSSAP00000019027	−1.155
	sialic acid-binding Ig-like lectin 14	ENSSSAG00000053773	NA*	NA	ND
lgals8b	galectin 8b	ENSSSAG00000015506	NA*	NA	ND
colec11	collectin subfamily member 11	ENSSSAG00000007870	NA*	NA	ND
CLEC19A	C-type lectin domain containing 19A	ENSSSAG00000009890	NA*	NA	ND
clec19a	C-type lectin domain containing 19A	ENSSSAG00000000444	NA*	NA	ND
	fucolectin-6-like	ENSSSAG00000044745	NA*	ENSSSAP00000040748	NS

Red, ASG-10 lower than gill; green, ASG-10 higher than gill; NS in grey, unchanged; NA* denotes genes expression detected in ASG-10 only. Log2fc, Log 2-fold change; ND, not detected; NA, not applicable.

#### 3.2.5 Immune responses (cytokines, receptors and innate immunity markers)

Eight interleukin (IL) genes were expressed in ASG-10, the majority of which (IL-34, pro-IL-16-like, IL-12 β subunit-like and IL-18) were detected at lower levels in the cells compared with gill (Log2fc between −4 and −1.6, Table 5). In contrast, IL-34-like and IL-8-like gene expression was detected at higher levels in the cells and additional IL-12 β-subunit-like and IL11a genes were expressed in ASG-10 only. Both mRNA and protein corresponding to Pro-IL-16 were detected. The Pro-IL-16 protein was present at similar levels in ASG-10 and gill. Expression of receptor subunits (mRNA only) for IL-1, IL-4, IL-6, IL-10, IL-11, IL-12, IL-13, IL-15, IL-17, IL-20 and IL-21 was found in ASG-10. Among these, IL-1 receptor type 1-like, IL-20 receptor subunit β-like and two IL-6 receptor β-like mRNA were detected at significantly higher levels in ASG-10 compared with gill. mRNA from three toll-like receptor (TLR) genes were detected in ASG-10, namely TLR-5 which was detected at higher levels in ASG-10 cells compared with gill (Log2fc 2.8), as well as TLR-18 and TLR2 type-2, both of which were found in the cells only. The corresponding proteins were not found in the proteomics dataset. Nucleotide-binding oligomerization domain-containing (NOD) 1 and 2 mRNAs were detected in ASG-10, both lower in the cells compared with gill, however NOD-2 mRNA was found in ASG-10 cells only. Interestingly, expression of 2 suggested pan B-cell marker genes: IL-22-like and IL79A-like were detected in the ASG-10 cell line, albeit at lower levels compared to gill. Antimicrobial peptide (AMP) mRNAs were also present in ASG-10. Cathelicidin mRNA was detected at a lower level in the cells (Log2fc −4.2), as was NK-lysin mRNA (Log2fc −5.2), whereas hepcidin-1 mRNA was detected at slightly higher levels (Log2fc 1.3). AMP 2-like peptide mRNA, annotated as “liver-expressed”, was detected in ASG10 cells only. Other AMPs such as defensins, were not detected. Regarding other markers of innate immunity, expression levels of serum amyloid A-like and the pentraxin related C-reactive protein genes were similar in both cells and gill, whereas pentraxin-related PTX-3-like mRNA was detected in ASG-10 cells only (data not shown).

**TABLE 5:** Profile of immune gene expression and proteins detected in ASG-10 relative to gill. Log2fc for genes expressed with significant differences: −0.3 to −12.7 (lower); 0.2 to 9.0 (higher).

*Gene name*	*Gene description*	*Gene ID*	*Gene Log2fc*	*Protein ID*	*Protein Log2fc*
Interleukins					
il34	Interleukin 34	ENSSSAG00000052184	−4.004	NA	ND
	pro-interleukin-16-like	ENSSSAG00000054402	−3.539	ENSSSAP00000057112	NS
IL12B	interleukin-12 subunit beta-like	ENSSSAG00000009655	−3.177	NA	ND
il18	*Salmo salar* Interleukin-18 (il18), mRNA.	ENSSSAG00000068522	−1.164	NA	ND
il34	interleukin-34-like	ENSSSAG00000069254	1.37	NA	ND
	interleukin-8-like	ENSSSAG00000052334	1.375	NA	ND
	interleukin-12 subunit beta-like	ENSSSAG00000068948	NA*	NA	ND
il11a	interleukin 11a	ENSSSAG00000041570	NA*	NA	ND
Interleukin Receptors					
il15ra	interleukin 15 receptor subunit alpha	ENSSSAG00000057898	−1.366	NA	ND
il17rd	interleukin 17 receptor D	ENSSSAG00000007352	−4.251	NA	ND
	interleukin-1 receptor type 1-like	ENSSSAG00000069897	1.349	NA	ND
	interleukin-10 receptor subunit beta-like	ENSSSAG00000074738	−4.229	NA	ND
I10R2	interleukin-10 receptor subunit beta-like	ENSSSAG00000049854	−2.429	NA	ND
il11ra	interleukin-11 receptor subunit alpha-like	ENSSSAG00000047554	NS	NA	ND
	interleukin-11 receptor subunit alpha-like	ENSSSAG00000004944	−0.955	NA	ND
IL12B	interleukin-12 subunit beta-like	ENSSSAG00000009655	−3.177	NA	ND
il13ra2	interleukin-13 receptor subunit alpha-2-like	ENSSSAG00000051928	−3.233	NA	ND
il13ra2	interleukin-13 receptor subunit alpha-2-like	ENSSSAG00000067691	−3.172	NA	ND
	interleukin-17 receptor E-like	ENSSSAG00000006676	−4.342	NA	ND
	interleukin-20 receptor subunit beta-like	ENSSSAG00000046741	3.054	NA	ND
il21r.1	interleukin-21 receptor-like	ENSSSAG00000055593	−4.869	NA	ND
il12rb2l	interleukin-6 receptor subunit beta-like	ENSSSAG00000043192	−6.233	NA	ND
il6st	interleukin-6 receptor subunit beta-like	ENSSSAG00000062955	1.598	NA	ND
	interleukin-6 receptor subunit beta-like	ENSSSAG00000059232	−1.319	NA	ND
	interleukin-6 receptor subunit beta-like	ENSSSAG00000004269	0.878	NA	ND
i12r2	*Salmo salar* Interleukin-12 receptor beta-2 chain (i12r2), mRNA.	ENSSSAG00000042273	−4.665	NA	ND
i13r2	*Salmo salar* Interleukin-13 receptor alpha-2 chain (i13r2), mRNA.	ENSSSAG00000081176	−0.879	NA	ND
il4ra	*Salmo salar* Interleukin-4 receptor alpha chain (il4ra), mRNA.	ENSSSAG00000040597	−2.011	NA	ND
TLRs					
TLR5	toll like receptor 5	ENSSSAG00000045202	2.774	NA	ND
tlr18	toll-like receptor 18	ENSSSAG00000010267	NA*	NA	ND
tlr18	toll-like receptor 18	ENSSSAG00000071033	NA*	NA	ND
AMPs					
	*Salmo salar* cathelicidin antimicrobial peptide (LOC100136439), mRNA.	ENSSSAG00000049319	−4.177	NA	ND
nkl	*Salmo salar* Antimicrobial peptide NK-lysin (nkl), mRNA.	ENSSSAG00000009346	−5.171	NA	ND
hamp1	hepcidin-1	ENSSSAG00000068171	1.283	NA	ND
	liver-expressed antimicrobial peptide 2-like	ENSSSAG00000018662	NA*	NA	ND
NOD					
nod1	nucleotide-binding oligomerization domain-containing protein 1-like	ENSSSAG00000064543	−4.256	NA	ND
nod1	nucleotide-binding oligomerization domain-containing protein 1-like	ENSSSAG00000053537	−3.923	NA	ND
nod2	nucleotide-binding oligomerization domain containing 2	ENSSSAG00000076025	−0.496	NA	ND
nod2	nucleotide-binding oligomerization domain containing 2	ENSSSAG00000043772	NA*	NA	ND
B-Cell Markers					
	B-cell receptor CD22-like	ENSSSAG00000001154	NS	NA	ND
igbp1	immunoglobulin (CD79A) binding protein 1	ENSSSAG00000069164	−1.378	NA	ND
igbp1	*Salmo salar* immunoglobulin (CD79A) binding protein 1 (igbp1), mRNA.	ENSSSAG00000068773	−0.56	NA	ND

Red, ASG-10 lower than gill; green, ASG-10 higher than gill; NS in grey, unchanged; NA* denotes genes expression detected in ASG-10 only. Log2fc, Log 2-fold change; ND, not detected; NA, not applicable.

#### 3.2.6 Adherent and tight junctions

Adherent junction and tight junction proteins are integral to the function of epithelial cells. It was therefore important to evaluate their presence in ASG-10 at both RNA and protein level and their variation between the cells and gill. Supplementary Table S2 shows the overview of the tight junction mRNAs and proteins including claudin, occludin, integrin and the major adherent junction transmembrane protein e-cadherin mRNAs and proteins detected in both ASG-10 and gills. Expression of 117 adherent and tight junction genes were detected and most were found at a higher level in ASG-10 compared with gill (transcripts from 42 genes [36%]). From these, 38 genes (32%) were expressed at a lower level, with a lower number expressed at a similar level (transcripts from 10 genes [9%]). Twenty-seven proteins (23%) were detected only in ASG-10. A total of 67 cadherin genes were expressed in ASG-10. The majority of those were detected at a higher level in the cells compared with gill and 22 were detected in ASG-10 only. Cadherin-13-like and two β-cadherin-like proteins were found in the corresponding proteomics dataset and all 3 were detected at a similar level in both cells and gill. A small number of claudin genes (11) were expressed in ASG-10. Of these, most were expressed at a higher level in the ASG-10 cells and 3 were expressed in ASG-10 only. There were no corresponding claudin proteins detected in the proteomics dataset. A larger number of integrin genes (35) were found expressed in ASG-10 and the majority of these (19) were expressed at a lower level in the cells. The corresponding protein for integrin β-2, integrin α-1-like, integrin β-1-like and integrin subunit β-4 were found in the proteomics dataset. Only integrin subunit β-4 was expressed at a slightly lower level in the cells. All other integrin proteins were detected at a similar level in cells and gill. Transcription of integrin-β-8 was detected only in the ASG-10 cells. Transcription of only 3 zonulae occluding (ZO) genes were found in the data, all coding for a ZO2-like protein and all were detected at a lower level in ASG10 compared with gill. However, the corresponding ZO2 protein (ENSSSAP00000088444), had a modest positive Log2fc of 1.4. Only one occludin gene was found expressed in ASG10 only.

#### 3.2.7 Biotransformation

The P450 genes expressed were mainly from the cyp2 family, including cyp2K1-like, cyp2F2 and cyp2M1, which were detected in ASG-10 only. However, levels of cyp1A1 differed most between the cells and gill and were considerably lower in ASG-10. Other cyp mRNAs detected at a lower level in the cell line included: cyp4, cyp27 and cyp120 (Table 6). Transcription levels of cyp 20A1-like and cyp3A27-like were similar in ASG-10 and gill. Cyp genes expressed at a higher level in ASG-10 were of different types, with 2 cyp2, 2 cyp51, a cyp20 and a cyp11 family member included in this cohort. The majority of phase II biotransformation genes expressed in ASG-10 (Table 6) were from glutathione S-transferase (GST) (10), followed by uridine 5′-diphospho-glucoronyltransferase (UGT; 4) and finally cytosolic sulfotransferase (SULT; 1). The ugt1a1 gene was expressed at a modestly lower level in the cells compared with gill. Its corresponding protein was also detected at a moderately lower level in the cells (Log2fc −0.7). Transcription of ugt5g2 and ugt1b7 was detected in ASG-10 only. Transcription of only 1 SULT gene was detected in ASG-10 cells, namely cytosolic sulfotransferase 3 (st1s3). The corresponding protein was also detected. Ten GST genes were also expressed in the cell line with 4 at a lower level, 5 at a similar level and 1 expressed at a modestly higher level in the cell line compared with gill. Three GST proteins were detected in the proteomic analysis, namely GST theta-1-like, GST omega-2 and GST-P. GST theta-1-like protein was present at a similar level in both cells and gill. GST omega-2 and GST-P proteins were present at a modestly lower level in ASG-10 cells (Log2fc of −1.0 and −0.8 respectively).

**TABLE 6:** Profile of biotransformation gene expression and proteins detected in ASG-10 relative to gill: Log2fc for genes expressed with significant differences: −0.3 to −12.7 (lower); 0.2 to 9.0 (higher).

*Gene name*	*Gene description*	*Gene ID*	*Gene Log2fc*	*Protein ID*	*Protein Log2fc*
Cytochrome P450 (phase I)					
cyp2k1	cytochrome P450 2K1-like	ENSSSAG00000003945	NA*	NA	ND
Cyp2f2	cytochrome P450 2F2-like	ENSSSAG00000065958	NA*	NA	ND
Cyp2m1	cytochrome P450 2M1-like	ENSSSAG00000069683	NA*	NA	ND
cyp1a	*Salmo salar* cytochrome P450 1A	ENSSSAG00000071998	−4.385	NA	ND
Cyp2k1	cytochrome P450 2K1	ENSSSAG00000039646	−3.279	NA	ND
cyp2ad2	cytochrome P450, family 2, subfamily AD, polypeptide 2	ENSSSAG00000067784	−2.933	NA	ND
cyp2u1	cytochrome P450 2U1	ENSSSAG00000049238	−2.683	NA	ND
CYP2U1	cytochrome P450 family 2 subfamily U member 1	ENSSSAG00000004992	−1.667	NA	ND
Porb	P450 (cytochrome) oxidoreductase b	ENSSSAG00000075049	−1.448	NA	ND
cyp2ae1	cytochrome P450, family 2, subfamily AE, polypeptide 1	ENSSSAG00000044739	−1.409	NA	ND
cyp4t8	cytochrome P450 4B1-like	ENSSSAG00000070173	−1.316	NA	ND
cyp27c1	cytochrome P450 27C1	ENSSSAG00000008167	−1.048	NA	ND
	putative cytochrome P450 120	ENSSSAG00000075162	−0.790	NA	ND
cyp20a1	cytochrome P450 20A1-like	ENSSSAG00000046176	NS	NA	ND
cyt3a27	cytochrome P450 3A27-like	ENSSSAG00000009258	NS	NA	ND
	cytochrome P450 2K1-like	ENSSSAG00000039163	0.444	NA	ND
cyp51	cytochrome P450, family 51	ENSSSAG00000005253	1.547	NA	ND
cyp20a1	cytochrome P450 20A1-like	ENSSSAG00000076973	1.673	NA	ND
cyp11a2	cytochrome P450, family 11, subfamily A, polypeptide 2	ENSSSAG00000045080	1.817	NA	ND
cyp51	cytochrome P450, family 51	ENSSSAG00000059749	1.834	NA	ND
Pora	cytochrome P450 oxidoreductase a	ENSSSAG00000066835	2.170	NA	ND
cyp2p6	cytochrome P450, family 2, subfamily P, polypeptide 6	ENSSSAG00000010049	3.781	NA	ND
	cytochrome P450 2D15-like	ENSSSAG00000009321	5.867	NA	ND
SULT, GST, UGT (phase II)					
ugt5g2	UDP glucuronosyltransferase 5 family, polypeptide G2	ENSSSAG00000059171	NA*	NA	ND
ugt1b7	UDP glucuronosyltransferase 1 family, polypeptide B7	ENSSSAG00000059820	NA*	NA	ND
st1s3	*Salmo salar* Cytosolic sulfotransferase 3	ENSSSAG00000078689	−5.241	ENSSSAP00000112282	−0.9
GSTT1	glutathione S-transferase theta-1-like	ENSSSAG00000069741	−6.117	ENSSSAP00000087456	NS
mgst1.1	microsomal glutathione S-transferase 1-like	ENSSSAG00000040892	−3.432	NA	ND
Gstcd	glutathione S-transferase, C-terminal domain containing	ENSSSAG00000052843	−0.682	NA	ND
	glutathione S-transferase kappa 1-like	ENSSSAG00000007549	−0.552	NA	ND
ugt1a1	UDP-glucuronosyltransferase-like	ENSSSAG00000080390	−0.532	ENSSSAP00000117144	−0.7
ugt5f1	UDP glucuronosyltransferase 5 family, polypeptide F1	ENSSSAG00000006447	−0.802	NA	ND
gsto2	glutathione S-transferase omega 2	ENSSSAG00000076348	NS	ENSSSAP00000105574	−1.0
MGST3	microsomal glutathione S-transferase 3-like	ENSSSAG00000074236	NS	NA	ND
GSTP1	glutathione S-transferase P	ENSSSAG00000003722	NS	ENSSSAP00000007559	−0.8
GSTT1	glutathione S-transferase theta-1-like	ENSSSAG00000064478	NS	NA	ND
MGST3	microsomal glutathione S-transferase 3-like	ENSSSAG00000003545	NS	NA	ND
gstz1	glutathione S-transferase zeta 1	ENSSSAG00000004511	0.508	NA	ND

Red, ASG-10 lower than gill; green, ASG-10 higher than gill; NS in grey, unchanged; NA* denotes gene expression detected in ASG-10 only. Log2fc, Log 2-fold change; ND, not detected; NA, not applicable.

#### 3.2.8 ABC transporters

Transcription of 35 genes across ABC transporter families A to F was detected in ASG-10. Transcription of approximately half (17 genes) were detected at either a lower or moderately lower level (Log2fc <1) in ASG-10, with the remainder detected at a higher or similar level. Three ABC transporter genes were expressed in ASG-10 only (Table 7). Only one ABC transporter, namely ABCF1, was detected in the proteomics dataset and was present at a lower level in the cells compared with gill. Solute carrier organic-anion transporters including slcob2a1, 3a1, 4a1 and 5a1 were found in the transcriptomics dataset (Table 7). Transcription of slco1c1 was detected in ASG-10 only. It is worth noting that the presence of PGP/ABCB1 mRNA or protein was note detected in the cells. This corresponds with the Calcein-AM assay and the PGP western blot (Figure 6). Two MRP4/ABCC4 genes were expressed at a lower level in ASG-10 cells compared with gill. There was no evidence of the corresponding proteins in the proteomics dataset, which also aligns with the Calcein-AM assay data (Figure 6).

**TABLE 7:** Profile of ABC and solute organic/anion transporter gene expression detected in ASG-10 relative to gill. The protein was present at a lower level in ASG-10 (Log2fc = −1.7). Log2fc for genes expressed with significant differences: −0.3 to −12.7 (lower); 0.2 to 9.0 (higher).

*Gene name*	*Gene description*	*Gene ID*	*Gene Log2fc*
ABC transporters			
abca12	ATP-binding cassette, sub-family A (ABC1), member 12	ENSSSAG00000057999	−9.765
abcg2	*Salmo salar* ATP binding cassette subfamily G member 2)	ENSSSAG00000042683	−4.490
abca1a	ATP-binding cassette sub-family A member 1-like	ENSSSAG00000006489	−3.714
abcc6a	ATP-binding cassette, sub-family C (CFTR/MRP), member 6a	ENSSSAG00000072785	−2.513
abcc3	ATP-binding cassette, sub-family C (CFTR/MRP), member 3	ENSSSAG00000069554	−1.971
ABCC4	multidrug resistance-associated protein 4-like	ENSSSAG00000081257	−1.943
abcc5	multidrug resistance-associated protein 5-like	ENSSSAG00000071602	−1.355
abca2	ATP-binding cassette, sub-family A (ABC1), member 2	ENSSSAG00000048721	−1.136
abcd3b	ATP-binding cassette sub-family D member 3-like	ENSSSAG00000017253	−1.111
abcc4	multidrug resistance-associated protein 4-like	ENSSSAG00000081593	−1.097
abcd3a	ATP-binding cassette sub-family D member 3-like	ENSSSAG00000056965	−0.792
abcb6a	ATP-binding cassette sub-family B member 6, mitochondrial-like	ENSSSAG00000007343	−0.791
abcd3b	ATP-binding cassette sub-family D member 3-like	ENSSSAG00000057454	−0.712
abcd1	ATP-binding cassette, sub-family D (ALD), member 1	ENSSSAG00000047976	−0.653
abcd3a	ATP-binding cassette, sub-family D (ALD), member 3a	ENSSSAG00000067766	−0.514
abcb10	ATP-binding cassette, sub-family B (MDR/TAP), member 10	ENSSSAG00000072382	−0.424
abcc10	ATP-binding cassette, sub-family C (CFTR/MRP), member 10	ENSSSAG00000016022	−0.396
abcf3	ATP-binding cassette sub-family F member 3-like	ENSSSAG00000070340	NS
ABCD2	ATP-binding cassette sub-family D member 2-like	ENSSSAG00000068252	NS
abcb8	ATP-binding cassette sub-family B member 8, mitochondrial-like	ENSSSAG00000048268	NS
abcb9	ATP-binding cassette, sub-family B (MDR/TAP), member 9	ENSSSAG00000080327	NS
abcb7	ATP binding cassette subfamily B member 7	ENSSSAG00000001480	NS
ABCD2	ATP-binding cassette sub-family D member 2-like	ENSSSAG00000052715	NS
abcc2	ATP binding cassette subfamily C member 2	ENSSSAG00000080954	NS
abce1	ATP-binding cassette, sub-family E (OABP), member 1	ENSSSAG00000075686	NS
abcf1 **	*Salmo salar* ATP-binding cassette, sub-family F (GCN20), member 1	ENSSSAG00000073367	0.881
ABCE1	ATP binding cassette subfamily E member 1	ENSSSAG00000074487	1.003
abcf2	*Salmo salar* ATP-binding cassette, sub-family F (GCN20), member 2	ENSSSAG00000054770	1.185
abcd4	ATP-binding cassette, sub-family D (ALD), member 4	ENSSSAG00000062923	1.259
abcc10	multidrug resistance-associated protein 7-like	ENSSSAG00000057074	1.584
abcb6a	ATP-binding cassette sub-family B member 6, mitochondrial-like	ENSSSAG00000059269	3.140
Solute organic/anion transporters			
slco1c1	solute carrier organic anion transporter family member 1C1-like	ENSSSAG00000074331	NA*
slco2b1	solute carrier organic anion transporter family member 2B1-like	ENSSSAG00000039031	−3.028
slco2b1	solute carrier organic anion transporter family member 2B1-like	ENSSSAG00000053908	−2.270
	canalicular multispecific organic anion transporter 2-like	ENSSSAG00000055677	−1.200
slco5a1	solute carrier organic anion transporter family member 5A1-like	ENSSSAG00000067369	−0.917
slco3a1	solute carrier organic anion transporter family, member 3A1	ENSSSAG00000039779	NS
slco3a1	solute carrier organic anion transporter family member 3A1-like	ENSSSAG00000074765	NS
slco5a1	solute carrier organic anion transporter family member 5A1-like	ENSSSAG00000078521	0.896
slco4a1	solute carrier organic anion transporter family member 4A1-like	ENSSSAG00000063615	0.955
slco5a1	solute carrier organic anion transporter family member 5A1-like	ENSSSAG00000072039	1.283

Red, ASG-10 lower than gill; green, ASG-10 higher than gill; NS in grey, unchanged; NA* denotes genes expression detected in ASG-10 only, **, the only ABC and solute organic/anion transporter protein detected was encoded by gene abcf1. Log2fc, Log 2-fold change; ND, not detected; NA, not applicable.

## 4 Discussion

Here, we further characterize the Atlantic salmon gill cell line, ASG-10, presented initially by Gjessing and co-workers (Gjessing et al., 2018). That initial study captured certain key characteristics of the newly developed cell line, including migratory and proliferation ability, cell morphology (consistent with epithelial cells), confirmation of Atlantic salmon origin, and virus susceptibility. To increase our understanding of the biology of ASG-10 cells, we generated a transcriptomic and proteomic profile of the cells and compared their expression patterns to native Atlantic salmon parr gills. Additionally, *in vitro* biophysical characteristics typical of epithelial cells, namely barrier function, ion transporters, phagocytosis, biotransformation and ABC transporters were explored. To our knowledge, this is the first time that such a comprehensive approach has been taken to examine a cell line derived from fish.

Here, like in other multi-omics studies on cells (Liu et al., 2016), we observed a difference in the sensitivity of the transcriptomics (22726 genes) compared with the proteomic analysis (2,376 proteins). This is a typical challenge of multi-omics analyses and has led to some of the discrepancies in the comparison between gene and protein expression data. Whereas the proteomic data represented primarily highly expressed proteins with housekeeping functions, the transcriptomic data also revealed differences in mRNA molecules present at low levels (>10 reads included in the data set). This was further reflected in an analysis of KEGG terms, which demonstrated the clear dominance of basic cellular processes, including metabolic pathways as well as biosynthetic pathways, ribosomal and spliceosome activity for proteins in all comparisons between ASG-10 cells and gill, whereas the transcriptome data revealed other pathways. KEGG analysis of the genes expressed at lower levels in ASG-10 highlighted a lower level of necroptosis in ASG-10 cells compared with gill. This controlled cell death pathway has elements of both apoptosis and necrosis (Dhuriya and Sharma., 2018) and its increased occurrence in the gill may be due to cell damage during excision and harvesting of the gills, post-mortem. Similarly, the lower identification of cell signaling pathways such as VEGF, MAPK signaling as well as the phosphatidylinositol signaling system are likely a result of the lack of crosstalk between different cell types in the ASG-10 cell culture compared with gill. The lower level of immune gene mRNAs reflected the likely lack of immune cells in the culture.

### 4.1 Biophysical characteristics

#### 4.1.1 ASG-10 homogeneity

Staining for goblet and chloride cells indicated that neither cell type was present in the cell culture and that ASG-10 most likely is a pure culture of pavement epithelial cells. The fundamental epithelial characteristics of the ASG-10 cells are reflected throughout the study. Reflecting the lack of chloride cells present in the ASG-10 cell line, there was an absence of NCC transcripts. However, transcription of a number of other osmoregulatory genes including some Cl/HCO3- anion exchanger, NHE3, V-type H^+^ ATPase, and NKA genes, were detected at a similar or higher level in ASG-10 cells. These genes, while normally associated with chloride cells, (Christensen et al., 2018), are not exclusively expressed by them. For example, the Cl/HCO3- anion exchanger, Slc4a1-3 variants as well as SLC26a 2,5,6 and 9 variants detected in ASG-10 are known to be expressed in both chloride and epithelial cells (Romero et al., 2013). A low number of mucin genes were expressed in ASG-10, including a number of mucin-2 and 5-ac genes which encode secreted, gel-forming mucins, known to be expressed in Atlantic salmon gill (Sveen et al., 2017). Additionally, transcription of membrane bound or associated mucin genes, mucin-13-like, 17-like and 18-like was also detected in the cells. mRNA levels of these genes in the cells compared with gill varied, however the apparently lack of goblet cells in the ASG-10 cell line was reflected in the small number of mucins detected. Interestingly, the detection of slightly higher expression levels of the mucin-17-like gene may be associated with the higher proliferation rate of the cells in culture, relative to the natural proliferation rate of the gill epithelial cells. In accordance with this, it was shown that cells proliferating at a high rate, such as human cancer cells, have an increase in expression of muc-17 (Hirono et al., 2010).

#### 4.1.2 Phagocytic activity

Epithelial cells are known to act as “non-professional” phagocytes, particularly involved in the ingestion and recycling of other apoptotic epithelial cells (Günther and Seyfert., 2018) in a process known as efferocytosis. As part of an organism’s first line of defense, epithelial cells engulf pathogens through stimulation of cytoskeletal and membrane re-arrangement by the pathogens, causing uptake and subsequent destruction via phagolysosomes. While phagocytosis by fish gill epithelial cells is not well studied, there is evidence that the shellfish gill epithelium is capable of bacterial phagocytosis *in vivo,* as demonstrated in mussels (Tame et al., 2022), and *in vitro,* as shown with gill epithelial cell primary cultures from abalone (Pichon et al., 2013). Therefore, the phagocytic ability of ASG-10 was examined and, true to their nature, they successfully took up fluorescently labelled bioparticles, derived from fungal cells (Zymosan - *S. cerevisiae -*insoluble β-1,3-glucan polysaccharide) and bacterial cells (*E. coli*). Differences were observed in the efficiency of the bioparticle uptake, with the *E. coli* phagocytosed more readily than the Zymosan- *S. cerevisiae* counterpart. This was reflected in the expression of receptors involved in the phagocytic process. The Dectin 1 receptor, a C-type lectin receptor which is known as the main phagocytic receptor for Zymosan in mammals, is not found in salmon (Petit et al., 2019). In a recent paper attempting to identify the Zymozan binding receptor in carp, a C-type lectin domain 4 gene was detected among several candidate genes (Petit et al., 2019). Several C-type lectin domain 4 genes were transcribed at a lower level in ASG-10 compared to the gill, which putatively accounts for the lack of efficient Zymosan uptake. Phagocytosis of bacteria partly depends on the mannose receptor. High levels of mannose receptor C type 2 mRNA were detected in ASG-10, higher than in gill samples. Galectins are also involved in bacterial recognition, (Patel et al., 2020), and several galectin family members were detected in ASG-10. The detection of high levels of flagellin-binding TLR5 transcripts may also further induce bacterial phagocytosis. Expression of clathrin and dynamin genes in ASG-10 was comparable to that in gill. Both clathrin and dynamin are central to clathrin-mediated endocytosis which was the proposed mechanism of nanoparticle uptake in RTgill-W1 (Felix et al., 2017). Therefore, it is plausible that similar experiments modelling nanoparticle uptake would be possible with ASG-10.

#### 4.1.3 Immune responses (cytokines, receptors and innate immunity markers)

The KEGG term “immune and inflammatory response” was clearly associated with the genes expressed at lower levels in ASG-10 compared to the gill. Transcripts from only a few genes in ASG-10 were associated with pathogen recognition pathways mediated by TLRs, NOD-like receptors and C-type lectins. This is likely due to the lack of immune cells, such as dendritic cells, macrophages and neutrophils, in the culture. More specifically, many TLRs detected in the gills were not detected in the ASG-10 transcriptome or proteome. Among them are the viral RNA receptors TLR-3, -7, -8 and -22, (data not shown), which indicates that ASG-10 cells may not be suitable for studying antiviral responses). This lack of virus sensing receptors may also be a reason for the viral susceptibility of the cell line, as demonstrated by Gjessing and co-workers (Gjessing et al., 2018), who showed that ASG-10 were susceptible to infectious hematopoietic necrosis virus (IHNV), viral hemorrhagic septicemia virus (VHSV), infectious pancreatic necrosis virus (IPNV), Atlantic salmon reovirus TS (TSRV) and Pacific salmon paramyxovirus (PSPV). In contrast, relatively high expression levels of the bacterial flagellin receptor TLR-5 were detected in ASG-10 cells, which makes them interesting for bacterial response studies (Günther and Seyfert, 2018). In line with this, an IL-8-like gene was expressed at higher levels in ASG-10 cells compared with gill. The expression of IL-8 is known to be induced through interaction of bacteria with TLRs (Eckman et al., 1993). Of note, mRNA from 2 TLR-18 genes, a teleost specific TLR, were detected in ASG-10 cells only. This TLR was previously confirmed to be expressed in gills of Atlantic salmon and its gene expression response to both viral and parasitic infection in head kidney has been reported (Lee et al., 2014). Its presence in ASG-10 cells provides the opportunity to use the cell line as a model to further investigate the role of TLR18 in gills. IL-22 and Il79A have been suggested as pan-B-cell markers in salmon (Peñaranda et al., 2019), but were detected at low levels in the ASG-10 cells, which could draw their specificity for B-cells in doubt. The IL11a cytokine was detected in ASG-10 only. Interestingly, IL11a has been characterized in trout, and found to be induced in gills upon bacterial infection (Wang et al., 2005). Transcription of AMPS, cathelicidin and NK-lysin was detected at lower levels in ASG-10 cells compared with gill. Expression of these genes is inducible by microbial and parasitic infection, therefore their presence at a lower level in the unchallenged cell line is likely to be expected (Acosta et al., 2019). Despite this, their presence does provide a baseline from which to assess their response as part of an infection challenge model incorporating ASG-10 cells.

#### 4.1.4 Adherent and tight junctions

Adherent and tight junctions are the linchpin of the selectively permeable barrier formed by epithelial cell layers. These junctions are formed by the interaction of several proteins including ZO proteins, occludins, claudins, integrins and cadherins. In gill epithelia of teleost fish, tighter junctions are associated with freshwater fish compared with “leaky” junctions associated with marine fish (Chasiotis et al., 2012). In line with this, the data presented here clearly demonstrated the ability of the freshwater gill-derived ASG-10 cells to express tight junction genes and to form tight junctions. The increased TER corresponds to the positive staining of ZO-1 protein between cells as a function of time, again confirming that the ASG-10 cells are epithelial. These data were complemented by the evidence of an overall higher expression of tight junction genes in ASG-10 cells compared to gill. However, the barrier generated by the ASG-10 might be considered as leaky as the ZO-1 staining pattern is somewhat punctuated and the tightness of the barrier, expressed as TER, is considerably lower than primary cells (Bury et al., 2014).

#### 4.1.5 Biotransformation

The variation in cyp gene mRNA levels in ASG-10 compared with gill pointed to a differential ability to metabolize both exogenous and endogenous substrates, including a range of fatty acids and drugs. However, this variability was likely due to the inherently inducible nature of these genes (Manikandan Nagini, 2018). Clearly, the gill, having been sourced from a living organism producing a variety of metabolites and exposed to various environmental stressors had a different cyp expression profile compared with a cell line grown in stable conditions (FBS supplemented media). For example, while the cyp1A1 gene was expressed at a considerably lower level in the cell line (Log2fc −4.4), exposure of the ASG-10 cells to β-naphthoflavone, a known inducer of CYP1A (Heinrich et al., 2014), induced both CYP1A protein expression and activity (Ivanova et al., 2023). Also, the gill cell line LG-1 from Atlantic Lumpfish (*Cycloperus lumpus L)* was found to have inducible CYP1A activity (Sindre et al., 2021), while the gill cell line from rainbow trout (RTgill-W1) and Waking catfish (*Clarias batrachus*), GB1, had no detectable CYP1A activity after β-naphthoflavone treatment (Franco et al., 2018). Interestingly, while expression of several of the cyp genes such as cyp1A1 is well characterized in Atlantic salmon gill (Olsvik et al., 2015; Alderman et al., 2020), many of the cyp genes emerging from the transcriptomics data are reported here in Atlantic salmon gill for the first time. These includes cyp2k1, cyp2D15-like and cyp2p6 which are detected at higher levels in ASG-10 compared with gill. Quantitative PCR studies evaluating the expression of cyp2k1 in Coho and Chinook salmon found that the gene was expressed hepatically but was not evident in the gill (Lavado and Aparicio fabreschleng., 2014, Matsuo et al., 2008). The cyp2D15 gene has primarily been characterized in dogs, (van Hagen et al., 2020). Cyp2p genes appear to be teleost specific and have so far been characterized in mangrove killfish, channel catfish and zebrafish (Goldstone et al., 2010; Zhang et al., 2014; Lee et al., 2018). Their presence and expression level, as well as the transcription of all reported cyp genes in ASG-10 provide the opportunity to expand toxicological studies in fish cell lines to include these genes/proteins and their substrates and therefore, increase our knowledge of gill biotransformation ability.

Sulfotransferase enzymes are responsible for the sulfation of a variety of compounds including xenobiotics and endogenous materials such as carbohydrates and proteins. Cytosolic sulfotransferase (SULT) enzymes are a subfamily involved in the biotransformation of hydrophobic environmental xenobiotics (Suiko et al., 2017). Transcripts from the cytosolic sulfotransferase gene *st1s3* and its corresponding protein were detected at a lower level in ASG-10 cells compared with gill. Again, as with cyp gene expression, this is likely due to lack of exposure of the naïve cells to external stimulants. However, the overall expression level of this gene at both RNA and protein level in the cell line would indicate its potential *in vitro* utility as a biomarker of phase II biotransformation response. Such work would be insightful as, to date, SULT enzyme expression and activity in salmon and associated cell lines have only been explored in a limited number of studies (Gonzalez et al., 2009; Barry and Marwanunez, 2010; Srikanth et al., 2017). Glutathione S-transferases (GSTs) are well known for removing environmental pollutants and endogenous toxic compounds as part of the phase II detoxification process through glutathionylation of diverse electrophilic substrates (Sukhovskaya et al., 2017). They have been suggested biomarkers for oxidative stress after exposure of polycyclic aromatic hydrocarbons (Santana et al., 2018). Taking a broad view of the transcription levels of UGT and GST genes detected in this study, it can be concluded that these levels were similar in both the cell line and the gill. Significant differences in expression, when observed, were modest (<1) for the majority of the genes in question. This was also true for UGT and GST genes whose corresponding protein expression levels were detected. Of note, expression of the *gstt1* gene was detected at a considerably lower level in ASG-10 cells compared with the gill (Log2fc −6). Any future work utilizing the expression of these genes as putative biomarkers should take this into consideration when using the cell line. However, like cyp and sulfotransferase, these genes are inducible. Additionally, the protein level of the corresponding GST theta-1 like enzyme was not significantly different in the cells compared with gill. This provides another example of how the transcriptomic and proteomic data do not always align due to the offset of mRNA levels compared with protein levels in the cell (Liu et al., 2016).

#### 4.1.6 ABC transporters

Aquatic organisms deploy a wide range of protective systems against harmful substances in water. Organs with blood-barrier or excretion functions, such as brain, liver and kidney possess a variety of membrane transporters, which are responsible for cellular efflux of endogenous substrates, as well as xenobiotics and their metabolites. A prominent family of membrane transporters are the ATP-binding cassette (ABC) transporters (Ferreira et al., 2014; Luckenbach et al., 2014) as well as the solute organic/anion transporters (OATs), which, together with drug metabolizing enzymes and transmembrane transporters, are important determinants of drug metabolism and drug clearance. The Calcein-AM assay using the probenecid inhibitor of MRP/organic-anion transporters showed a significant increase in Calcein-AM accumulation, indicating the presence of corresponding transporters in the cells. This correlates with the data showing that transcription of organic-anion transporter genes slco3a1, 4a1 and 5a1 were detected at a similar or moderately increased level in the ASG-10 cells compared with gill. However, the well-characterized ABC transporter ABCB1, also known as PGP1, was not expressed in ASG-10 as shown by the lack of response of the PCS833 inhibitor and the failure to detect the PGP protein in the cells with western blotting. It should be noted that PGP was not found in the transcriptomic or proteomic profile of gill. Taken together, this lack of expression is consistent with findings in other fish species (Ferreira et al., 2014). There have been limited studies of ABC transporter activity in fish gills. A study by (Kropf et al., 2020) compared ABC transporter expression in Rainbow trout gill with a primary trout gill cell culture. As in our study, transcription of abcg2, abcc2, abcc3 genes was detected. Importantly, the expression pattern of the transporters differed in the primary culture compared with gill, which is similar to the findings in our study. For example, mRNA molecules from the lipid transporter abca12, xenobiotic transporter abcg2, and the cholesterol efflux transporter abca1 are detected at higher level (Log2fc>3) in gill, whereas the heavy-metal and porphyrin transporter abca12 was detected at a higher level in ASG-10 cells. As suggested by Kropf et al. (2020), these differences could be attributed to media composition and the two-dimensional nature of cell culture compared to the three-dimensional nature of a gill.

## 5 Conclusion

Our broad characterization of ASG-10 supports the suitability of the cell line for a range of *in vitro* experimental studies to better understand gill functions, including epithelial regeneration and barrier function, drug biotransformation, transport and clearance, ion channel activity, studies of metabolism, phagocytic function, and host/pathogen interaction - in particular for bacteria. The ASG-10 cell line can further promote development of a functional gill model. Co-cultures with other cell types and fluidic organ-on-a-chip systems using ASG-10 can be developed in the future to create a more realistic gill model, and to better represent the complexity of the gills.

## Data Availability

The raw data supporting the conclusions of this article will be made available by the authors, without undue reservation. The RNA-seq (transcriptomic) data has been uploaded to NCBI SRA with the accession number PRJNA1004542. The mass spectrometry proteomics data have been deposited to the ProteomeXchange Consortium via the PRIDE (Perez-Riverol et al., 2022) partner repository with the dataset identifier PXD044533.
