# Supplementary material for: Functional and molecular characterization of the Atlantic salmon gill epithelium cell line ASG-10; a tool for in vitro gill research
Source: Front Mol Biosci. 2023 Oct 17;10:1242879. doi: 10.3389/fmolb.2023.1242879 (PMC10616884; doi:10.3389/fmolb.2023.1242879)
Supplement: Supplementary file 1 [file Table1.docx]

Supplementary Material

**Functional and molecular characterization of the Atlantic salmon gill epithelium cell line ASG-10 as a tool for *in vitro* gill research**

**Orla Slattery, Maria K Dahle, Arvind Y.M. Sundaram, Barbara F. Nowak, Mona C. Gjessing and Anita Solhaug***

*** Correspondence:** [Anita.Solhaug@vetinst.no](mailto:Anita.Solhaug@vetinst.no)

**Supplementary Table S1:** Summary of transcriptomic data analysis

**Supplementary Table S2:** Profile of adherent and tight junction gene expression and proteins detected in ASG-10 relative to gill**.**

**Supplementary Figure S1**: GO analysis of genes and proteins expressed at similar, higher and lower levels in ASG-10 compared with gill.

**Supplementary Figure S2.** KEGG analysis of genes (A) and proteins (B) expressed at similar levels (no significant differences), and proteins at significantly lower levels in ASG-10 compared with gill (C).

**Supplementary Table S1:** Summary of transcriptomic data analysis. Abbreviations: raw_read_pairs = number of sequenced read pairs; clean_read_pairs = number of reads passing filter and pre-processing; hista2_uniqe_align_fragments = number of read pairs that are aligned to a unique location in the Altantic genome using HiSAT2; featureCounts_unique_assigned_fragments = number of read pairs that were counted for further analysis. These read pairs have aligned to the gene region in the right orientation.

| **Sample Name** | **Raw read pairs** | **Clean read pairs** | **hisat2 unique align fragments** | **Feature Counts unique assigned fragments** |
| --- | --- | --- | --- | --- |
| Gill_1 | 34,317,522 | 33,702,871 | 24,612,835 | 14,568,669 |
| Gill_2 | 39,849,269 | 39,432,484 | 29,109,035 | 19,311,363 |
| Gill_3 | 39,159,096 | 38,826,856 | 28,822,250 | 19,316,453 |
| Gill_4 | 43,816,193 | 42,988,678 | 32,081,801 | 20,880,604 |
| Gill_5 | 43,101,347 | 42,725,434 | 32,554,943 | 22,273,479 |
| ASG10_ 1 | 35,331,712 | 34,690,953 | 26,321,475 | 20,807,562 |
| ASG10_ 2 | 39,945,824 | 39,640,351 | 31,924,095 | 25,435,909 |
| ASG10_ 3 | 52,911,480 | 52,520,455 | 43,219,963 | 32,427,962 |
| ASG10_ 4 | 42,536,719 | 42,283,162 | 33,700,032 | 24,940,299 |
| ASG10_ 5 | 49,788,443 | 49,412,674 | 39,889,875 | 31,390,573 |

**Supplementary Table S2:** Profile of adherent and tight junction gene expression and proteins detected in ASG-10 relative to gill. Red = ASG-10 lower than gill, green = ASG-10 higher than gill, grey = unchanged. Log2fc = Log 2-fold change. ND= not detected. NA = not applicable. Log2fc for genes expressed with significant differences: -0.3 to -12.7 (lower); 0.2 to 9.0 (higher). Log2fc for proteins with significant differences: -0.8 (lower); 1.4 (higher).

| ***Gene name*** | ***Gene description*** | ***Gene id*** | ***Log2fc*** | ***Protein ID*** | ***Protein Log2fc*** |
| --- | --- | --- | --- | --- | --- |
| **Claudins** | | | |  |  |
| cldn5b | claudin 5b | ENSSSAG00000052084 | -4.404 | NA | ND |
| cldn1 | claudin-1-like | ENSSSAG00000038146 | -4.384 | NA | ND |
| cld19 | claudin-19-like | ENSSSAG00000079973 | -0.288 | NA | ND |
| Cldnd | Salmo salar claudin d | ENSSSAG00000063722 | 0.702 | NA | ND |
| cldnd1b | claudin domain-containing protein 1-like | ENSSSAG00000004512 | 0.876 | NA | ND |
| cldnd1b | claudin domain-containing protein 1-like | ENSSSAG00000041100 | 1.056 | NA | ND |
| cld12 | Salmo salar Claudin-12 | ENSSSAG00000047812 | 1.187 | NA | ND |
| cldn12 | claudin 12 | ENSSSAG00000046665 | 2.333 | NA | ND |
| **Integrins** | | | |  |  |
| itgae | integrin subunit alpha E | ENSSSAG00000076346 | -9.423 | NA | ND |
| itgam | integrin alpha-X-like | ENSSSAG00000047200 | -7.92 | NA | ND |
| itb2 | Salmo salar Integrin beta-2 | ENSSSAG00000067161 | -7.165 | ENSSSAP00000080482 | NS |
| itga1 | integrin alpha-1-like | ENSSSAG00000040699 | -5.981 | ENSSSAP00000033658 | NS |
| itga1 | integrin alpha-1-like | ENSSSAG00000007494 | -4.747 | NA | ND |
| itga3 | integrin subunit alpha 3 | ENSSSAG00000053873 | -4.53 | NA | ND |
| itgbl1 | integrin, beta-like 1 | ENSSSAG00000044364 | -4.483 | NA | ND |
| itga5 | integrin alpha-5-like | ENSSSAG00000064017 | -3.571 | NA | ND |
| itga8 | integrin, alpha 8 | ENSSSAG00000003604 | -3.571 | NA | ND |
| itgb2 | integrin beta-2-like | ENSSSAG00000022772 | -2.475 | NA | ND |
| itgb1a | integrin beta-1-like | ENSSSAG00000039442 | -2.18 | NA | ND |
| itga2.2 | integrin alpha-2-like | ENSSSAG00000010010 | -1.861 | NA | ND |
| itga11b | integrin, alpha 11b | ENSSSAG00000065857 | -1.861 | NA | ND |
| itgb3a | integrin beta-3-like | ENSSSAG00000001418 | -1.748 | NA | ND |
| ITGA6 | integrin alpha-6-like | ENSSSAG00000053761 | -1.575 | NA | ND |
| itgav | integrin subunit alpha V | ENSSSAG00000075460 | -1.55 | NA | ND |
| itga9 | integrin, alpha 9 | ENSSSAG00000003063 | -1.501 | NA | ND |
| itgb1a | integrin, beta 1a | ENSSSAG00000080301 | -1.458 | NA | ND |
| itgb5 | integrin beta-5-like | ENSSSAG00000080528 | -1.458 | NA | ND |
| ITGA7 | integrin alpha-7-like | ENSSSAG00000058863 | NS | NA | ND |
| itfg2 | integrin alpha FG-GAP repeat containing 2 | ENSSSAG00000055802 | NS | NA | ND |
| itga4 | integrin subunit alpha 4 | ENSSSAG00000064161 | NS | NA | ND |
| itga4 | integrin alpha 4 | ENSSSAG00000078815 | NS | NA | ND |
| itga2b | integrin alpha-IIb-like | ENSSSAG00000051488 | NS | NA | ND |
| itfg1 | integrin alpha FG-GAP repeat containing 1 | ENSSSAG00000063909 | 0.695 | NA | ND |
| itga5 | integrin alpha-5-like | ENSSSAG00000076696 | 1.017 | NA | ND |
| ITGB1 | integrin beta-1-like | ENSSSAG00000007621 | 1.214 | ENSSSAP00000036336 | NS |
| itgb5 | integrin beta-5-like | ENSSSAG00000067105 | 1.717 | NA | ND |
| Itga5 | integrin alpha-5-like | ENSSSAG00000007556 | 1.803 | NA | ND |
| ITGA7 | integrin alpha-7-like | ENSSSAG00000073644 | 2.077 | NA | ND |
| itga8 | integrin, alpha 8 | ENSSSAG00000042854 | 2.184 | NA | ND |
| itgb4 | integrin subunit beta 4 | ENSSSAG00000065365 | 2.827 | ENSSSAP00000076304 | -0.807 |
| itga9 | integrin, alpha 9 | ENSSSAG00000040941 | 3.223 | NA | ND |
| itga6b | integrin alpha-6-like | ENSSSAG00000066104 | 4.075 | NA | ND |
| **Zonula occludens** | | | |  |  |
| tjp2a | tight junction ZO-2-like | ENSSSAG00000081595 | -0.819 | NA | ND |
| tjp2b | tight junction ZO-2-like | ENSSSAG00000047300 | -1.101 | NA | ND |
| tjp2a | tight junction ZO-2-like | ENSSSAG00000063980 | -2.994 | ENSSSAP00000088444 | 1.408 |
| **Occludins** | | | |  |  |
| Ocln* | occludin | ENSSSAG0000076339 | NA* | NA | ND |
| **Cadherins** | | | |  |  |
| cdh26.2 | cadherin-13-like | ENSSSAG00000040049 | -8.013 | ENSSSAP00000031153 | NS |
| cdh5 | cadherin-5-like | ENSSSAG00000041358 | -4.295 | NA | ND |
| cdh11 | cadherin 11, type 2 | ENSSSAG00000057765 | -3.645 | NA | ND |
| bcdh | B-cadherin-like | ENSSSAG00000072016 | -3.322 | ENSSSAP00000094009 | NS |
| pcdh1b | protocadherin 1b | ENSSSAG00000002398 | -3.060 | NA | ND |
| cdh26.1 | cadherin26 tandem duplicate1 | ENSSSAG00000045657 | -2.622 | NA | ND |
| pchs17 | protocadherin 17 | ENSSSAG00000063294 | -1.724 | NA | ND |
| bcdh | B-cadherin-like | ENSSSAG00000063294 | -1.688 | ENSSSAP00000077718 | NS |
| dchs1a | protocadherin-16-like | ENSSSAG00000053291 | -1.274 | NA | ND |
| fat3a | FAT atypical cadherin 3a | ENSSSAG00000043150 | -1.194 | NA | ND |
| pcdh7b | protocadherin-7-like | ENSSSAG00000003088 | -0.947 | NA | ND |
| fat1a | protocadherin Fat 1-like | ENSSSAG00000042024 | -0.741 | NA | ND |
| pcdh18a | protocadherin-18-like | ENSSSAG00000041533 | -0.665 | NA | ND |
| fat3a | FAT atypical cadherin 3a | ENSSSAG00000059667 | -0.558 | NA | ND |
| fat2 | FAT atypical cadherin 2 | ENSSSAG00000057688 | NS | NA | ND |
|  | protocadherin Fat 4-like | ENSSSAG00000073671 | NS | NA | ND |
| cdh6 | cadherin-12-like | ENSSSAG00000042586 | NS | NA | ND |
| dchs1a | dachsous cadherin-related 1a | ENSSSAG00000051481 | NS | NA | ND |
|  | protocadherin gamma-C5-like | ENSSSAG00000042586 | 0.621 | NA | ND |
| fat1a | protocadherin Fat 1-like | ENSSSAG00000005406 | 0.661 | NA | ND |
|  | protocadherin alpha-C2-like | ENSSSAG00000002123 | 0.914 | NA | ND |
| pcdh15a | protocadherin-related 15a | ENSSSAG00000075648 | 0.947 | NA | ND |
| cdh11 | cadherin 11, type 2 | ENSSSAG00000041642 | 1.155 | NA | ND |
|  | protocadherin alpha-C2-like | ENSSSAG00000077194 | 1.168 | NA | ND |
| cdh10 | cadherin-10-like | ENSSSAG00000050663 | 1.446 | NA | ND |
| cdh6 | cadherin-6-like | ENSSSAG00000039218 | 1.466 | NA | ND |
| cdh2 | cadherin-2-like | ENSSSAG00000049983 | 1.603 | NA | ND |
|  | protocadherin-12-like | ENSSSAG00000076189 | 1.884 | NA | ND |
| cdh2 | cadherin-2-like | ENSSSAG00000079280 | 1.906 | NA | ND |
| pcdh15b | protocadherin-related 15b | ENSSSAG00000070424 | 2.181 | NA | ND |
|  | protocadherin alpha-4-like | ENSSSAG00000002164 | 2.319 | NA | ND |
|  | protocadherin gamma-C5-like | ENSSSAG00000002291 | 2.797 | NA | ND |
| pcdh7b | protocadherin-7-like | ENSSSAG00000040782 | 2.835 | NA | ND |
| pcdh7b | protocadherin-7-like | ENSSSAG00000040782 | 2.835 | NA | ND |
| cdh4 | cadherin-4-like | ENSSSAG00000081638 | 2.842 | NA | ND |
| cdh13 | cadherin-13-like | ENSSSAG00000069414 | 2.846 | NA | ND |
|  | protocadherin gamma-A12-like | ENSSSAG00000002528 | 2.992 | NA | ND |
| cdh4 | cadherin-4-like | ENSSSAG00000079678 | 3.15 | NA | ND |
| cdh4 | cadherin-4-like | ENSSSAG00000040968 | 3.552 | NA | ND |
| pcdh1g22 | protocadherin 1 gamma 22 | ENSSSAG00000077214 | 3.578 | NA | ND |
| pcdh18b | protocadherin-18-like | ENSSSAG00000003075 | 3.732 | NA | ND |
| CDH2 | cadherin-2-like | ENSSSAG00000079622 | 3.967 | NA | ND |
| cdhr5b | cadherin-related family member 5-like | ENSSSAG00000055278 | 3.976 | NA | ND |
| CDH2 | cadherin-2-like | ENSSSAG00000000678 | 4.251 | NA | ND |
| cdh15 | cadherin-15-like | ENSSSAG00000066810 | 4.590 | NA | ND |
| pcdhac2 | protocadherin alpha-C2-like | ENSSSAG00000002645 | 5.639 | NA | ND |


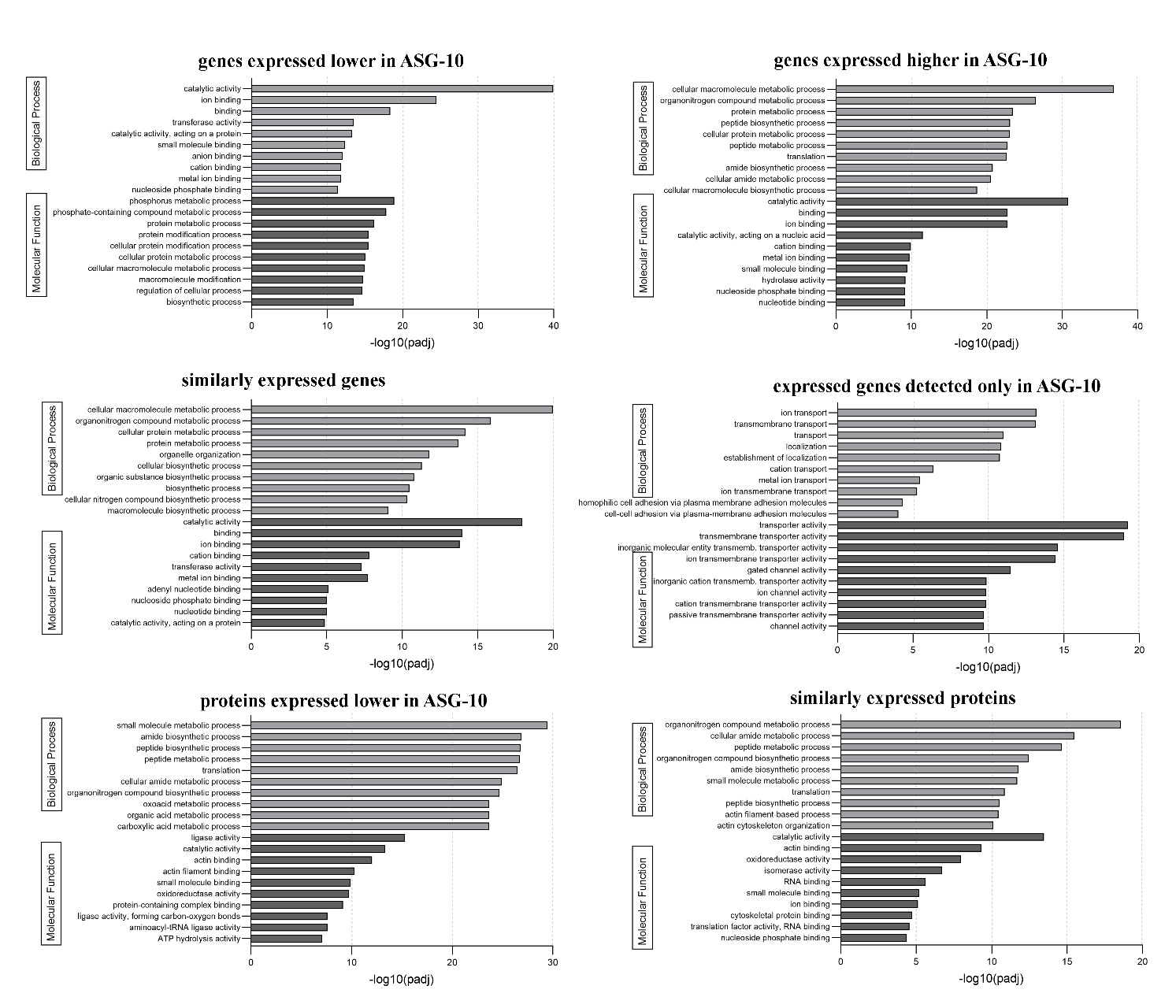


**Supplementary Figure S1**. GO analysis of genes and proteins expressed at similar, higher and lower levels in ASG-10 compared with gill. The top 10 molecular function and biological process terms that emerged from the analysis are presented.


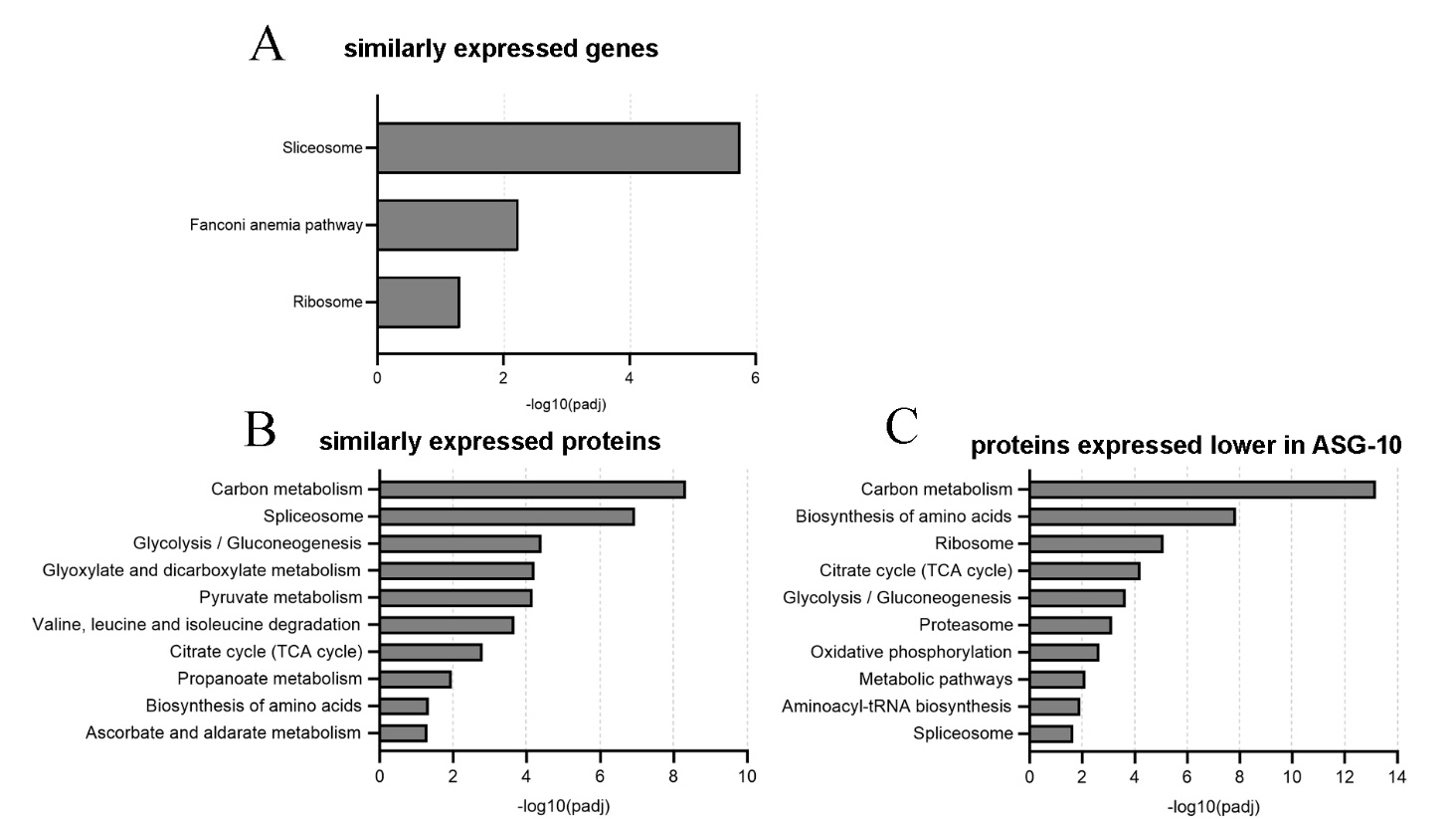


**Supplementary Figure S2.** KEGG analysis of genes (A) and proteins (B) expressed at similar levels (no significant differences), and proteins at significantly lower levels in ASG-10 compared with gill (C). The top 10 molecular function and biological process terms that emerged from the analysis are presented.

From the KEGG analysis of the genes expressed at a similar level in both ASG-10 and gills, only 3 terms emerged (Supplementary Figure 2A). Expression of genes in the Fanconi anaemia pathway (controlling repair of damaged DNA and expression of genes related to the spliceosome and ribosome appear to be consistent in both the cell line and gill. Proteins associated with metabolic pathways such as glycolysis and the TCA cycle were detected in the dataset of similarly expressed proteins as well as the dataset of protein expressed at a lower level in ASG-10 (Supplementary Figures 2A and B). The most dominant pathway common to both ASG-10 and gills is the citrate cycle (TCA cycle) and pathways closely associated/feeding into this cycle, including pyruvate metabolism, glyoxylate and dicarboxylate metabolism (which results in the production of oxaloacetate), as well as the val, leu and ile degradation pathways (which produces acetyl co-A and succinyl co-A) and the ascorbate and aldarate metabolism pathway (which involves the further metabolism of alpha-keto glutarate). In addition, the term “spliceosome” had the second highest enrichment score, indicating that proteins involved in mRNA splicing are expressed at a similar level in both ASG-10 and gills. Proteins involved in glycolysis and gluconeogenesis reactions appear to be expressed at similar levels in both ASG-10 and gill. When one examines the KEGG terms with a lower enrichment score, expression of proteins associated with glycolysis/gluconeogenesis, the TCA cycle, oxidative phosphorylation, biosynthesis of amino acids, ribosome and amino-acyl t-RNA synthesis, are lower in ASG-10 than in gills**.** Proteins associated with the proteasome (the multi-protein complex involved in degradation of cellular proteins) and some proteins associated with the spliceosome are also expressed at a lower level in ASG-10.

**
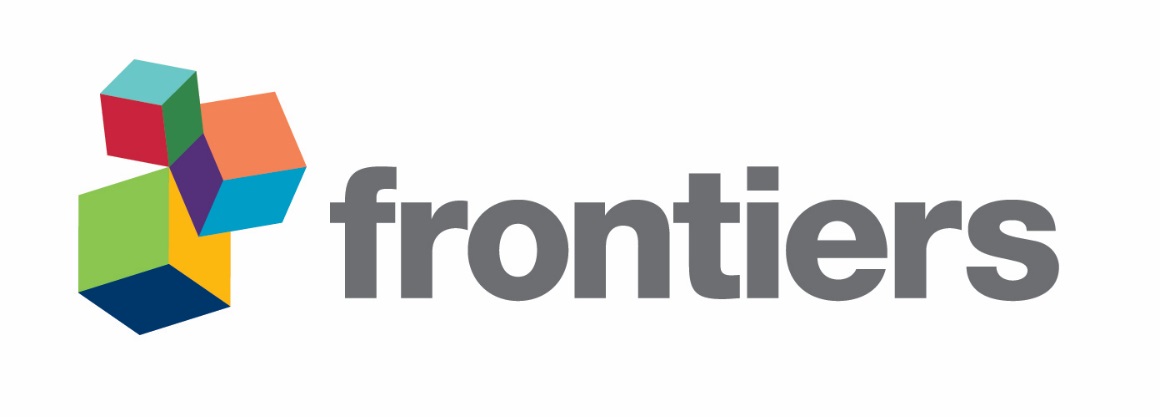
**
